# Ubiquitin Azapeptide Esters as Next-Generation Activity-Based Probes for Cysteine Enzymes in the Ubiquitin Signal Pathway

**DOI:** 10.1021/jacs.5c01732

**Published:** 2025-04-23

**Authors:** Saibal Chanda, Sandeep Atla, Xinlei Sheng, Satyanarayana Nyalata, Yugendar R. Alugubelli, Demonta D. Coleman, Wen Jiang, Rosana Lopes, Shaodong Guo, A. Joshua Wand, Yingming Zhao, Wenshe Ray Liu

**Affiliations:** † Department of Biochemistry and Biophysics, College of Agriculture and Life Sciences, 14736 Texas A&M University, College Station, Texas 77843, United States; ‡ Texas A&M Drug Discovery Center and Department of Chemistry, College of Arts and Sciences, 14736 Texas A&M University, College Station, Texas 77843, United States; § Ben May Department of Cancer Research, The University of Chicago, Chicago, Illinois 60637, United States; ∥ Department of Nutrition, College of Agriculture and Life Sciences, 14736 Texas A&M University, College Station, Texas 77843, United States; ⊥ Institute of Biosciences and Technology and Department of Translational Medical Sciences, School of Medicine, Texas A&M University, Houston, Texas 77030, United States; # Department of Cell Biology and Genetics, School of Medicine, 14736 Texas A&M University, College Station, Texas 77843, United States

## Abstract

Ubiquitination is a pivotal cellular process that controls protein homeostasis and regulates numerous biological functions. Its pathway operates through a cascade of enzyme reactions involving ubiquitin-activating (E1), ubiquitin-conjugating (E2), and ubiquitin-ligating (E3) enzymes and deubiquitinases (DUBs), many of which are cysteine enzymes. Activity-based ubiquitin probes were previously developed for profiling these enzymes. However, most conventional probes do not mimic natural enzyme–substrate interactions and involve chemical mechanisms different from enzyme catalysis. Their uses potentially affect the comprehensiveness of enzyme profiling results. The current study introduces a novel class of activity-based ubiquitin probes, ubiquitin azapeptide esters, designed to overcome these limitations. These probes incorporate an azaglycine ester at the ubiquitin *C*-terminus. They structurally mimic a ubiquitinated protein substrate and react with a cysteine enzyme via a mechanism like the enzyme catalysis. It was demonstrated that ubiquitin azapeptide esters are reactive toward a large variety of DUBs and several tested E1, E2, and E3 enzymes as well. Compared to a conventional probe, ubiquitin propargylamine, ubiquitin azapeptide esters generally provide superior labeling and profiling of active cysteine enzymes in the ubiquitination/deubiquitination cascade in both HEK293T cells and mouse tissue lysates. Activity-based protein profiling using these probes in mouse tissue lysates also revealed distinct patterns of labeled enzymes, confirming their potential in understanding the unique roles of these enzymes in different tissues.

## Graphical abstract

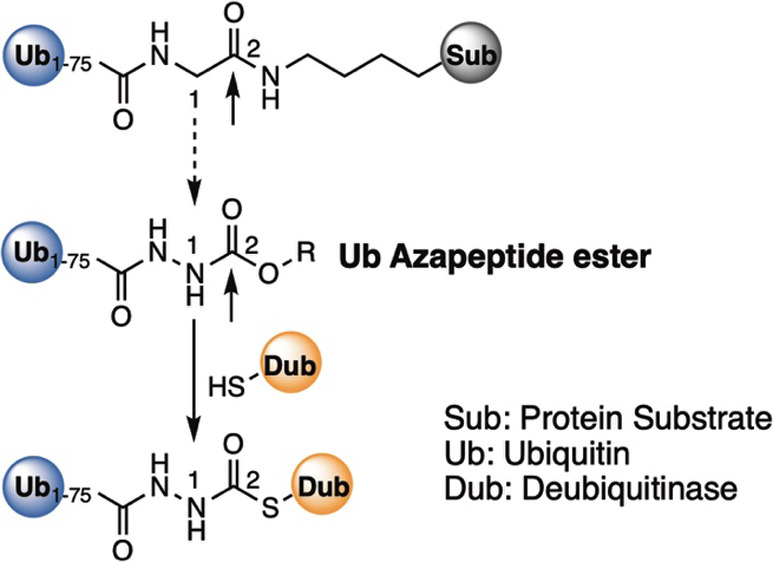

## Introduction

Ubiquitin is a small, evolutionarily conserved regulatory protein that is found in almost all eukaryotic organisms.^1^ It plays a crucial role in many cellular processes by attaching covalently to other proteins to regulate their function, location, or lifespan.^2^ This process is known as ubiquitination.^3^ Ubiquitination can lead to a variety of cellular outcomes. The most notable is to tag proteins for degradation by the proteasome.^4^ This mechanism, widely known as the ubiquitin-proteasome system, is essential for the regulation of many fundamental cellular processes.^5^ Due to its crucial role in maintaining protein homeostasis, the ubiquitin-proteasome pathway has implications in the pathogenesis of various diseases.^6–10^ As such, it is a target for therapeutic intervention. As shown in Figure 1A, protein ubiquitination operates through a highly regulated process involving a cascade of three groups of enzymes: E1 ubiquitin-activating enzymes, E2 ubiquitin-conjugating enzymes, and E3 ubiquitin ligases. Ubiquitination can stop at the attachment of a single ubiquitin (monoubiquitination) or continue to add a chain of ubiquitin molecules (polyubiquitination). This overall process is reversed by another group of enzymes, deubiquitinases (DUBs), that catalyze the hydrolysis of ubiquitin from a polyubiquitinated chain or substrate.

**Figure 1:**
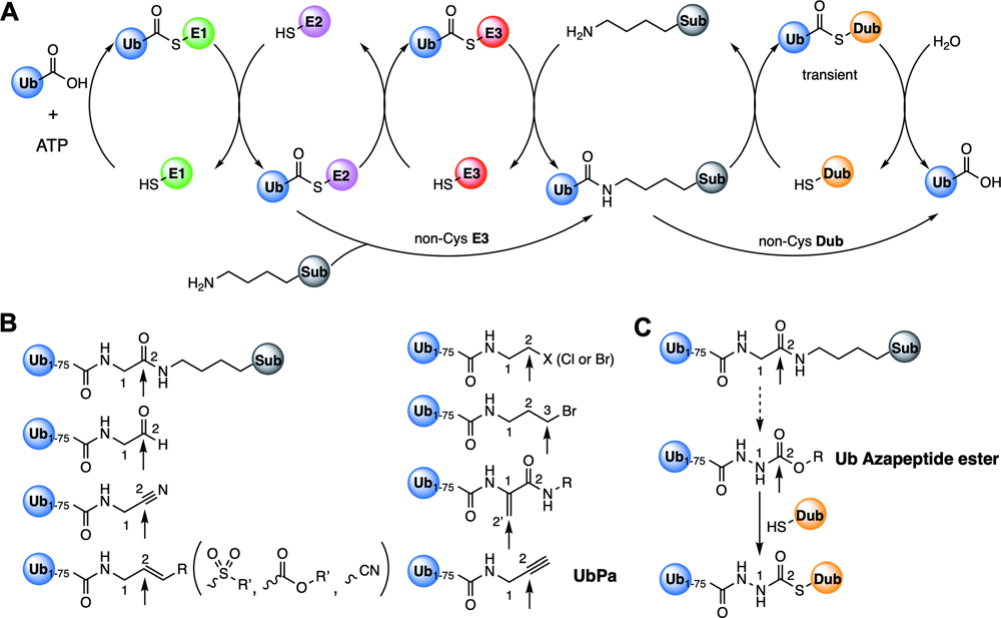
The ubiquitination/deubiquitination cascade and ubiquitin probes. (A) The E1–E2–E3 enzymatic cascade to covalently attach ubiquitin (Ub) to a protein substrate (Sub) and the reverse deubiquitination process catalyzed by deubiquitinase (DUB). Both E3 and DUB enzymes have two classes, either with a catalytic cysteine or without. Cysteine-based DUBs form covalent ubiquitin complexes only transiently that quickly hydrolyze. (B) Ubiquitin probes that have been developed so far for the study of enzymes in the ubiquitination/deubiquitination cascade. A ubiquitinated protein model is shown as a comparison. The ubiquitin Gly76 Cα and carboxyl carbon atoms are labeled as 1 and 2, respectively. The arrow pointing at 2 in Ub-Sub indicates the hydrolysis position that is also the site transiently conjugating with a catalytic DUB cysteine. Sites in a variety of probes that can covalently conjugate with cysteine enzymes in the ubiquitination/deubiquitination cascade are indicated with pointing arrows as well. (C) Proposed ubiquitin azapeptide esters that mimic a ubiquitinated protein (Ub-Sub) better than conventional ubiquitin probes for covalent conjugation with DUBs.

Among the four groups of enzymes in the ubiquitination/deubiquitination cascade, E1 and E2 enzymes are restrictedly cysteine enzymes, a small number of E3 enzymes are cysteine enzymes as well, and most DUB enzymes are cysteine proteases. All cysteine enzymes in this pathway form a covalent ubiquitin complex, either stable or transient, during their catalysis. This catalytic nature and the high nucleophilicity of their catalytic cysteine have led to the development of a variety of ubiquitin-based covalent probes for these enzymes, as shown in Figure 1B. The first ubiquitin probe is ubiquitin aldehyde that potently inhibits cysteine enzymes in the ubiquitination/deubiquitination cascade by forming a thiohemiacetal covalent adduct with the catalytic cysteine.^11,12^ Subsequently, another probe, ubiquitin nitrile, was synthesized as a potent inhibitor for an isopeptidase in the 26S proteasome by forming a thioimidate adduct with a cysteine.^13^ For these two probes, their covalent enzyme adducts are not stable and hydrolyze in water. For this reason, a series of ubiquitin probes that contain a Michael acceptor or an alkyl halide were synthesized. Reactions of cysteine enzymes with these probes form stable covalent adducts allowing even direct isolation of DUBs from cell lysates.^14^ A same purpose probe containing a dehydroalanine was developed later. Besides reacting with DUB enzymes, this ubiquitin probe was found to react with E1 and E2 enzymes as well.^15,16^ The currently most widely used covalent ubiquitin probe is probably ubiquitin propargylamine (UbPa).^17^ UbPa was synthesized by Ovaa et al. for a different purpose but found reactive toward DUBs efficiently. Additional covalent ubiquitin probes that react similarly to or with reaction mechanisms different from probes shown in Figure 1B have also been developed by Ploegh, Virdee, Zhuang, Brik, Shi, McGouran, Liu, Li, and their coworkers.^18–27^

A great application of covalent ubiquitin probes is their use to conduct activity-based protein profiling of cysteine enzymes in the ubiquitination/deubiquitination cascade. Activity-based protein profiling is a powerful technique developed by Cravatt and co-workers to study activities of a group of enzymes within complex biological systems.^28^ It is particularly useful for identifying active enzymes in a given sample.^29^ Applying this technique in combination with the use of different covalent ubiquitin probes has led to the elucidation of functionally active cysteine enzymes, mainly DUBs, in the ubiquitin cascade pathway in both tissues and disease cells.^18,21,30^ However, conventional ubiquitin probes, as shown in Figure 1B, are structurally different from a native ubiquitinated protein. Except for ubiquitin aldehyde that forms a thiohemiacetal adduct with an enzyme active site cysteine to mimic the enzyme’s transition state, all other probes react with the catalytic cysteine in enzymes through mechanisms different from enzymes’ catalytic mechanisms. Ubiquitin aldehyde cannot be used as an activity-based probe. Using other probes in the activity-based protein profiling analysis might potentially miss cysteine enzymes in the ubiquitination/deubiquitination cascade that require strict substrate binding and/or have an active site that doesn’t accommodate the transition state intermediate formed with a conventional ubiquitin probe. As such, we proposed ubiquitin azapeptide esters, shown in Figure 1C, as alternative activity-based probes for cysteine enzymes in the ubiquitination/deubiquitination cascade.

An azapeptide is a peptide molecule containing a nitrogen atom named as aza-nitrogen, in an original Cα position in the peptide backbone.^31^ This modification alters the electronic configuration of the peptide. An azapeptide ester has also its *C*-terminal side of aza-nitrogen converted to an ester. Compared to its parent peptide, an azapeptide ester is structurally highly similar and therefore maintains a similar binding pattern to a target enzyme. In addition, its ester as part of a carbamate group is chemically stable but reactive toward a cysteine protease to form a covalent adduct. Compared to the original amide bond, carbamate in azapeptide ester has a more electron deficient carbon that facilitates its reaction toward a nucleophile. Comparatively, the alkyne group in UbPa is relatively inert toward a nucleophile in physiological conditions. It has been shown that this covalent adduct is stable when the aza-amino acid is azaglycine, although other aza-amino acids will lead to a slowly hydrolyzing adduct.^32,33^ Since ubiquitin naturally has a *C*-terminal glycine, we envisioned that a ubiquitin azapeptide ester, as shown in Figure 1C, with its *C*-terminal glycine converted to an azaglycine ester would structurally and functionally mimic a ubiquitinated protein and therefore react with a cysteine-based DUB enzyme to form a stable covalent adduct that is a thiocarbamate. This azaglycine modification retains backbone flexibility similar to glycine, ensuring that a probe can still fit well within the narrow catalytic cleft of the enzymes. As shown in Figure S1A, a ubiquitin azapeptide ester will react with a DUB active site cysteine similarly as a ubiquitinated protein substrate to form a tetrahedral transition state, typical for acyl transfer reactions, that has an oxygen anion stabilized by the anion hole in the enzyme.^34^ Due to its better chemical reactivity toward a nucleophile than alkyne in UbPa, azapeptide ester in a ubiquitin probe is likely more potent toward some deubiquitinases. Due to its highly reactive nature toward an activated cysteine in an enzyme, we suspected that a ubiquitin azapeptide ester is likely active toward E1, E2, and cysteine-containing E3 enzymes as well by mimicking their substrates as shown in Figure S1B,C. Therefore, a ubiquitin azapeptide ester might be generally applied as an activity-based probe to profile cysteine enzymes in the ubiquitination/deubiquitination cascade. The reactivity of a ubiquitin azapeptide ester may also be tuned through the use of different leaving alcohol groups. This is easily achievable synthetically and provides a potentially additional advantage. In this work, we wish to show the development of ubiquitin azapeptide esters and their use as novel activity-based probes for mapping cysteine enzyme activities in the ubiquitination/deubiquitination cascade in cells and mouse tissues.

## Results and Discussion

### Synthesis of FlagUbPa and Three Ubiquitin Azapeptide Esters

The expressed protein ligation technique, in which a protein with intein fused at its *C*-terminus is recombinantly produced to form a protein thioester, has been most commonly used to generate a ubiquitin thioester for a subsequent reaction with a small molecule to afford a conventional ubiquitin probe.^35,36^ An alternative expressed protein ligation technique that requires no involvement of intein or any enzymes has also been developed and applied to the synthesis of ubiquitin and ubiquitin-like protein-small molecule conjugates.^37^ This technique, coined as activated cysteine-based protein ligation (ACPL), features a nitrile donor molecule, e.g., 2-nitro-5-thiocyanatobenzoic acid (NTCB), to activate a protein cysteine as shown in Figure 2A to form a 1-acyl-2-iminothiazolidine intermediate that subsequently undergoes a nucleophilic acyl substitution reaction with a small molecule amine. The overall reaction leads to replacing cysteine with the amine at the *C*-terminus of the target protein. Ubiquitin is naturally devoid of cysteine. We previously showed that using a recombinant approach to install a cysteine residue at its *C*-terminus and then conducting the ACPL reaction of the purified protein, a large variety of small molecule amines including different amino acids can be conjugated to the ubiquitin *C*-terminus.^38^ Due to its simplicity, we decided to use ACPL to generate ubiquitin azapeptide esters. Two recombinant ubiquitin proteins were expressed and purified from E. coli (Figure S2A). The first is Flag-Ub_1‑75_-Cys-6×His which has a G76C mutation followed by a 6×His tag and the second Flag-Ub_1‑74_-Cys-6×His which contains a G75C mutation followed by a 6×His tag. Both proteins have an *N*-terminal FLAG tag epitope (DYKDDDDK) for immunoprecipitation and Western blot analysis with an anti-FLAG antibody. Using a previously established ACPL protocol,^38^ we synthesized Flag-Ub_1‑75_-Pa (FlagUbPa) as a control probe by incubating Flag-Ub_1‑75_-Cys-6×His with tris­(2-carboxyethyl)­phosphine (TCEP), NTCB, and propargylamine. FlagUbPa was used during the whole study as a control molecule due to its wide use as an activity-based probe for DUB enzymes. Our proposed ubiquitin azapeptide esters that can serve as probes for cysteine enzymes in the ubiquitination/deubiquitination cascade are precisely Ub_1‑75_ azaglycine esters. They can be made from Flag-Ub_1‑75_-Cys-6×His by reacting it with different hydrazinecarboxylates. To tune reactivity in the final ubiquitin azapeptide esters, we initially designed three probes with methanol, ethanol, and trifluoroethanol leaving groups, respectively. As such, three hydrazinecarboxylates, methyl hydrazinecarboxylate (MTC), ethyl hydrazinecarboxylate (ETC), and 2,2,2-trifluoroethyl hydrazinecarboxylate were either acquired from commercial providers or synthesized for their reactions with Flag-Ub_1‑75_-Cys-6×His in the presence of TCEP and NTCB (Figure 2B). Using MTC and ETC led to successful synthesis of Flag-Ub_1‑75_-MTC (FlagUbMTC) and Flag-Ub_1‑75_-ETC (FlagUbETC), respectively. However, 2,2,2-trifluoroethyl hydrazinecarboxylate has low solubility in water, leading to a low reaction yield. As such, we synthesized 2,2,2-trifluoroethyl-2-glycylhydrazine-1-carboxylate (TFEGHC). TFEGHC is soluble in water. Reacting it with Flag-Ub_1‑74_-Cys-6×His in the presence of TCEP and NTCB afforded Flag-Ub_1‑74_-TFEGHC (FlagUbTFEGHC) (Figure 2B). Note that the three final ubiquitin azapeptide probes, FlagUbMTC, FlagUbETC, and FlagUbTFEGHC, differ only at their *C*-terminal alcohol leaving group. For FlagUbPa and three developed ubiquitin azapeptide probes, we isolated them using fast protein liquid chromatography (FPLC) to high purity as shown in Figure S3 and then analyzed them by electrospray ionization mass spectrometry (ESI-MS) analysis. The two original proteins, Flag-Ub_1‑75_-Cys-6×His and Flag-Ub_1‑74_-Cys-6×His were analyzed by ESI-MS as well for quality control. For all proteins, their original proton-charged and deconvoluted spectra are presented in Figures S4–S9. The deconvoluted ESI-MS spectra for the four final probes with their determined molecular weights indicated are also presented in Figure 2C. For all probes, their determined molecular weights match well with their theoretical values with a deviation of 0.3 Da (Table S1), confirming their successful synthesis.

**Figure 2:**
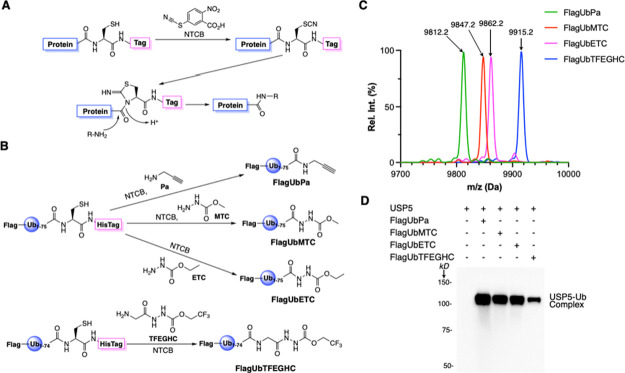
Activated cysteine-based protein ligation (ACPL) technique and its use in the synthesis of ubiquitin azapeptide ester probes. (A) Chemical mechanism of ACPL. A protein cysteine is activated by a nitrile donor molecule, e.g., 2-nitro-5-thiocyanatobenzoic acid (NTCB) to form a 1-acyl-2-iminothiazolidine intermediate that undergoes nucleophilic acyl substitution with an amine to generate a *C*-terminally functionalized protein. (B) Use of two ubiquitin proteins, Flag-Ub_1‑75_-Cys-6xHis and Flag-Ub_1‑74_-Cys-6xHis, to react with four amines including propargylamine (Pa), methyl hydrazinecarboxylate (MTC), ethyl hydrazinecarboxylate (ETC), and 2,2,2-trifluoroethyl-2-glycylhydrazine-1-carboxylate (TFEGHC), mediated by NTCB, to generate FlagUbPa and three ubiquitin azapeptide ester probes including FlagUbMTC, FlagUbETC, and FlagUbTFEGHC. (C) Deconvoluted electrospray ionization mass spectrometry (ESI-MS) spectra of four synthesized ubiquitin probes. Determined molecular weights match well with their calculated values. (D) Covalent conjugation of four ubiquitin probes with USP5. The generated USP5-Ub complexes are highlighted. Bovine serum albumin (BSA) was used as a loading control.

### Demonstrations of Ubiquitin Azapeptide Esters as Covalent Probes for Cysteine-Containing DUBs in the Ubiquitination/Deubiquitination Cascade

With the successful synthesis of three ubiquitin azapeptide esters, we proceeded to test their uses as covalent probes for cysteine enzymes in the ubiquitination/deubiquitination cascade. USP5 is a commercially available DUB enzyme that has a catalytic cysteine. We acquired this enzyme and conducted its reactions with FlagUbPa and three ubiquitin azapeptide esters. USP5 was incubated with four probes for 40 min at 37 °C and then analyzed by SDS-PAGE analysis followed by Western blotting with anti-FLAG. The result is presented in Figure 2D. USP5 itself has no FLAG tag, showing a blank lane in the Western blot image. On the contrary, its reactions with four probes led to the formation of a USP5-ubiquitin complex with a molecular weight above 100 kDa that was clearly detected by anti-FLAG. To ensure equal loading of both enzymes and probes, all reactions were conducted in a DUB reaction buffer containing bovine serum albumin (BSA) as a loading control that was detected using an anti-BSA antibody (Figure S10). For USP5, FlagUbMTC and FlagUbETC displayed reactivity like FlagUbPa. FlagUbTFEGHC showed weaker reactivity than the other three. Compared to methyl and ethyl groups in FlagUbMTC and FlagUbETC, respectively, the trifluoroethyl group in FlagUbTFEGHC is more electron-withdrawing. Therefore, chemically FlagUbTFEGHC is more reactive than FlagUbMTC and FlagUbETC. A likely reason for its comparatively lower reactivity is potential interference of the bulky trifluoroethyl group in the binding of FlagUbTFEGHC to USP5. Despite different reactivities that were observed, the results clearly demonstrated that all three ubiquitin azapeptide esters can serve as activity-based probes for cysteine-containing DUB enzymes.

Encouraged by the results with USP5, we expanded our investigation to evaluate the reactivity of three ubiquitin azapeptide esters across a diverse set of DUBs, all of which feature an active site cysteine. We obtained 16 DUBs, including eight from the USP family (USP2, USP7, USP8, USP10, USP14, USP15, USP25, and CYLD), one from the UCH family (UCHL3), two from the Josephin family (Ataxin-3 and JOSD2), and five from the OTU family (OTUB2, OTULIN, Cezanne-OTUD7B, OTUD2, and OTUD7C). Each enzyme was tested with the three ubiquitin azapeptide esters, with FlagUbPa serving as a control. Reactions of all DUBs with FlagUbPa and three ubiquitin azapeptide ester probes were conducted and analyzed in the same manner as for USP5. Molecular weights of each DUB and its covalent complexes with four probes are listed in Table S2A. As shown in Figure 3, tested DUB enzymes exhibited distinct reactivity profiles toward four probes. USP2 and UCHL3 showed comparable reactivity with all probes, displaying strong labeling efficiency (Figure 3A i,B). In contrast, USP7 demonstrated marked selectivity for FlagUbTFEGHC although overall labeling efficiency with all probes was strong (Figure 3A ii). The azapeptide ester probes exhibited higher reactivity compared to FlagUbPa with USP8, USP10, and USP25 (Figure 3A iii, iv, and vii). Both FlagUbMTC and FlagUbPa showed comparable reactivity toward USP14 (Figure 3A v), while FlagUbETC and FlagUbTFEGHC showed reduced reactivity. USP15 exhibited robust labeling with all four probes, with FlagUbPa being slightly more efficient than three azapeptide ester probes (Figure 3A vi). FlagUbPa and FlagUbTFEGHC exhibited similar reactivity toward CYLD, compared to the relatively weaker reactivity for FlagUbMTC and FlagUbETC toward this enzyme (Figure 3A viii). In the Josephin family, Ataxin-3 displayed a strong preference for ubiquitin azapeptide esters, whereas FlagUbPa exhibited minimal reactivity (Figure 3C i). For JOSD2, FlagUbMTC and FlagUbETC demonstrated covalent labeling comparable to FlagUbPa, while FlagUbTFEGHC showed reduced reaction (Figure 3C ii). The OTU family DUBs presented diverse covalent labeling patterns with four probes. FlagUbMTC and FlagUbETC exhibited enhanced reactivity with OTUB2 compared to FlagUbPa and FlagUbTFEGHC (Figure 3D i), while FlagUbMTC demonstrated superior labeling of OTULIN (Figure 3D ii), outperforming all other probes and suggesting it as an optimal ligand for this enzyme. Notably, FlagUbPa showed almost no reactivity toward OTULIN. For Cezanne-OTUD7B, all probes labeled the enzyme with similar high efficiency (Figure 3D iii). OTUD2 showed strong labeling with azapeptide ester probes, particularly with FlagUbMTC, while FlagUbPa displayed weak reactivity (Figure 3D iv). Interestingly, FlagUbPa failed to label OTUD7C, as indicated by the absence of a covalent complex band, where two of the azapeptide ester probes successfully engaged with the enzyme, underscoring potential advantage of ubiquitin azapeptide esters for enzymes with stringent substrate specificity (Figure 3D v). OTU DUBs often display isopeptide linkage selectivity during their catalyzed reactions.^39^ This higher reaction selectivity requirement likely contributes to their relatively better reactivity toward ubiquitin azapeptide ester probes.

**Figure 3:**
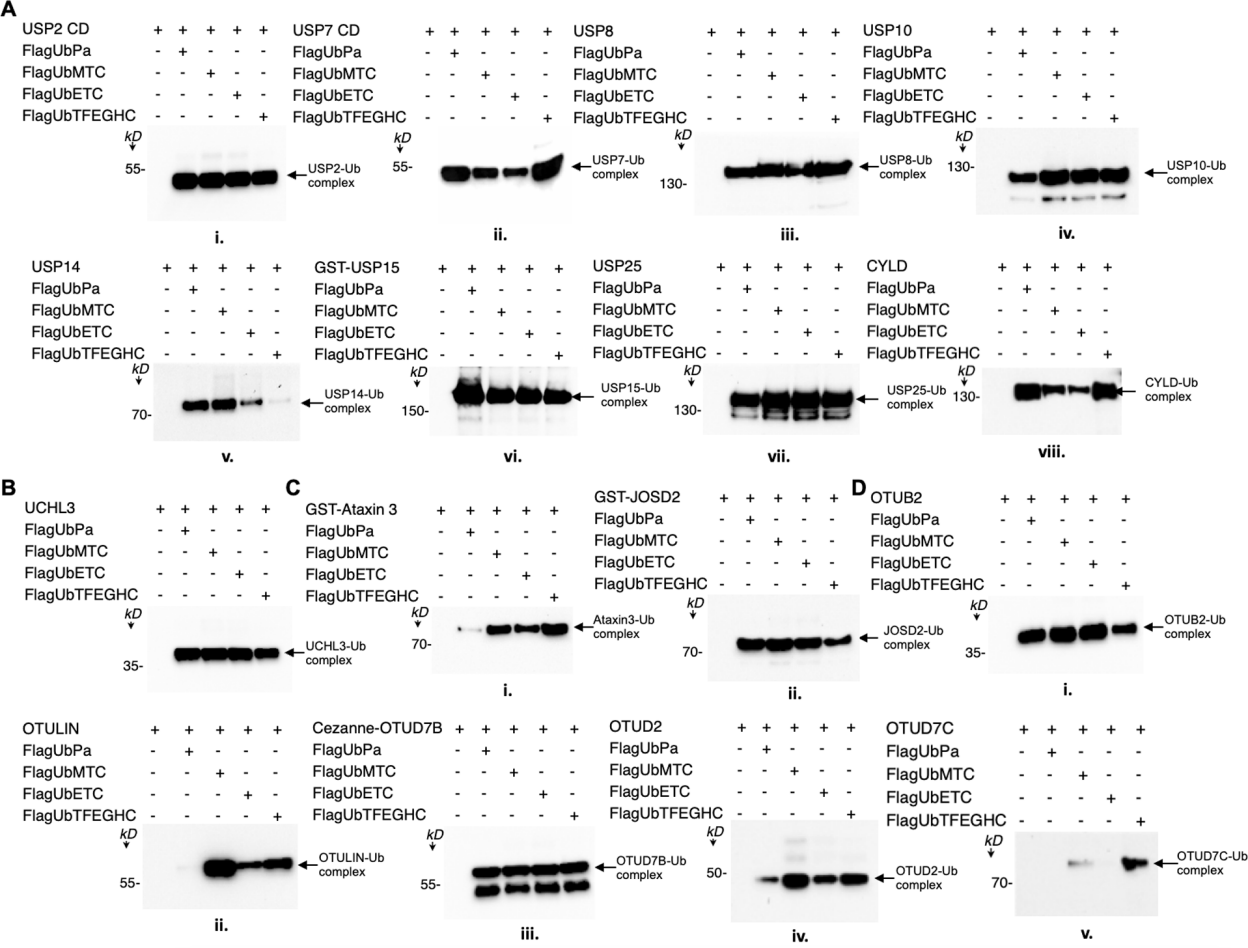
Ubiquitin azapeptide esters as covalent probes for cysteine-containing DUBs. DUBs were reacted with four probes at 37 °C for 40 min before they were analyzed by SDS-PAGE and probed by an anti-FLAG antibody. Covalent DUB-Ub complexes are indicated. BSA was used as a loading control for all reactions. (A) USP family DUBs including (i) USP2, (ii) USP7, (iii) USP8, (iv) USP10, (v) USP14, (vi) USP15, (vii) USP25, and (viii) CYLD. (B) A UCH family DUB - UCHL3. (C) Josephin family DUBs (i) Ataxin-3 and (ii) JOSD2. (D) OTU family DUBs including (i) OTUB2, (ii) OTULIN, (iii) Cezanne-OTUD7B, (iv) OTUD2, and (v) OTUD7C.

To rule out nonspecific covalent labeling of DUBs caused solely by the azapeptide ester group, we synthesized a short peptide, biotin-GG-LRG-MTC, which retained the *C*-terminal three residues of ubiquitin and azaglycine. This peptide was used to label all DUBs tested in Figures 2 and 3. The reactions were conducted under identical conditions, with the only modification being the substitution of a ubiquitin probe with biotin-GG-LRG-MTC. Due to the peptide’s low molecular weight (<1 kDa), the reaction mixtures were incubated with streptavidin before being analyzed by SDS-PAGE and probed using anti-streptavidin antibody via Western blotting. No covalent complex formation was observed between biotin-GG-LRG-MTC and any of the tested DUBs (Figure S12). These results indicated that, in the absence of the full ubiquitin scaffold to enhance binding, the azapeptide ester functional group alone is insufficient to induce covalent labeling of DUBs.

Data collected so far demonstrated that three ubiquitin azapeptide ester probes are generally better probes than FlagUbPa for covalent conjugation with DUBs. Except USP15 that displayed strong labeling with all probes among which FlagUbPa was slightly better, all tested DUBs exhibited reactivity comparable to or greater than FlagUbPa when tested with at least one azapeptide ester probe. Notably, certain OTU DUBs that were not labeled by FlagUbPa were effectively labeled by the azapeptide ester probes. OTU DUBs are known to recognize isopeptide linkages for more stringent substrate selectivity than USP DUBs. For OTULIN, OTUD2, and OTUD7C, their low to no labeling with FlagUbPa but efficient labeling with ubiquitin azapeptide ester probes are likely due to azapeptide ester probe’s resemblance to native ubiquitinated protein substrates. Low labeling of Ataxin 3 with FlagUbPa is likely due to the same reason. Therefore, applying ubiquitin azapeptide ester probes for activity-based protein profiling will likely reveal cysteine enzymes that are not targeted by FlagUbPa.

A selected group of E1, E2, and cysteine-containing E3 enzymes including UBE1, UBE2E2, Parkin, UBE3A, and ITCH-E3A were used to react with three ubiquitin azapeptide ester probes as well. As shown in Figure S13, these enzymes reacted with ubiquitin azapeptide ester probes with different preferences, supporting the notion that ubiquitin azapeptide ester probes may serve as activity-based probes for E1, E2, and cysteine-containing E3 as well.

### Formation and Stability of a DUB-Ub Thiocarbamate Covalent Complex

As shown in Figure 3, USP7 exhibited highly efficient and specific labeling with all three ubiquitin azapeptide ester probes. Therefore, we selected USP7 as a model to demonstrate formation and stability of a covalent DUB complex formed with a ubiquitin azapeptide ester probe. To synthesize the USP7-Ub covalent complex (Figure 4A), USP7 CD was incubated with FlagUbTFEGHC, with BSA used as a loading control (Figure S14). To assess whether complex formation increased over time, we performed a time-dependent assay by incubating USP7 CD with FlagUbTFEGHC for a total reaction time of 40 min at 37°C. At designated time points (0* [operation dead time <30 s], 5, 10, 20, and 40 min), the reaction was quenched by adding SDS-PAGE loading dye and subsequently analyzed. As shown in Figure 4B, the reaction reached completion by approximately 5 min. To evaluate the thermal stability, the USP7-Ub covalent complex was subjected to different temperatures (37, 45, 50, 55, and 60 °C) for 60 min. As shown in Figure 4C, the complex remained stable up to 45 °C but began to decompose at 50 °C and above. Next, we evaluated the stability of the USP7-Ub covalent complex over an extended period. The complex was incubated at 37 °C for up to 24 h. As shown in Figure 4D, the complex remained stable throughout the entire duration. Additionally, we examined the stability of the thiocarbamate bond in the presence of varying concentrations of glutathione using a thiol-thioester exchange assay conducted over 60 min. As shown in Figure 4E, except for a slight decrease in the Western blot signal at 10 mM glutathione, the complex remained stable across all tested concentrations. To confirm that formation of the USP7-Ub covalent complex requires an intact active-site cysteine, USP7 CD was pre-treated with 10 mM iodoacetamide before incubation with FlagUbTFEGHC. As shown in Figure 4F, iodoacetamide treatment completely inhibited complex formation, confirming that the USP7-Ub covalent complex was formed via a thiocarbamate linkage at the active-site cysteine. Based on these results, we conclude that ubiquitin azapeptide probes can form stable complexes with cysteine-containing enzymes through a thiocarbamate linkage. This stability, both over time and under varying conditions, supports their viability as effective activity-based probes for cysteine enzymes in the ubiquitination/deubiquitination cascade.

**Figure 4:**
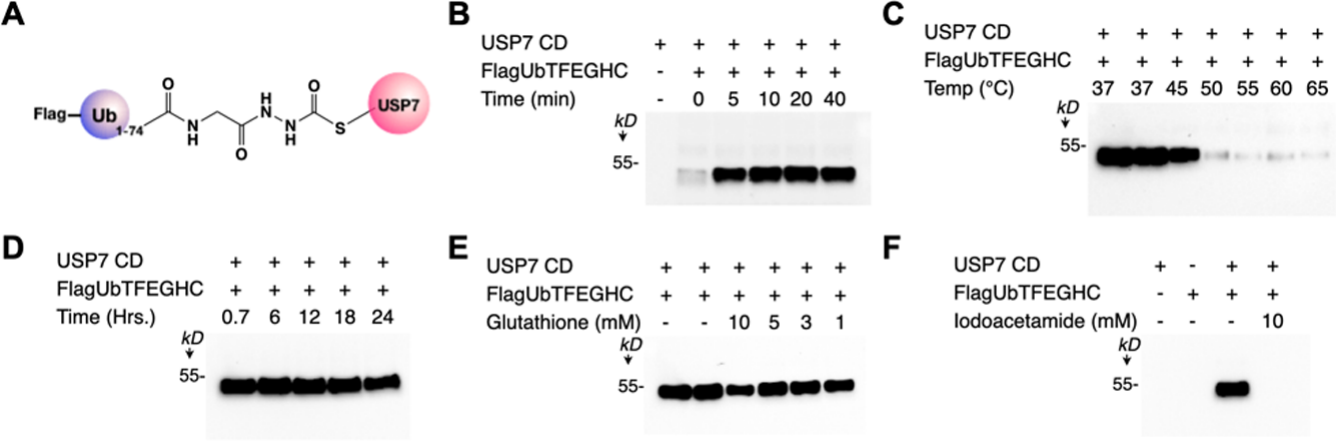
Characterization of a USP7-Ub thiocarbamate complex. (A) Structure of a USP7-Ub thiocarbamate complex generated from FlagUbTFEGHC and USP7 CD. (B) Time dependence of the complex formation at 37 °C. (C) Stability of the complex at different temperature for 60 min. (D) Stability of the complex over time at 37 °C. (E) Stability of the complex in the presence of varying concentrations of glutathione for 60 min. (F) Iodoacetamide blocks the complex formation.

### Activity-Based Protein Profiling of Cysteine Enzymes in the Ubiquitination/Deubiquitination Cascade Using Ubiquitin Azapeptide Ester Probes

To assess the ability of ubiquitin azapeptide esters in mapping cysteine enzyme activities in the ubiquitination/deubiquitination cascade, we performed labeling experiments of HEK293T cell lysates with three ubiquitin azapeptide esters. FlagUbPa was used as a control. To simplify the analysis, only the lysate supernatant was collected and used to react with four probes and same cell lysate samples were used for all four probes for every group of experiments to avoid potential variations between different batches of HEK293T cell lysates. We incubated the clarified supernatant with each of the four probes for 20 h at 4 °C before analyzing the reaction mixtures by SDS-PAGE and Western blot analysis with anti-FLAG. The result is presented in Figure 5A. Without adding a probe, there was only one visible band around 75 kDa detected by anti-FLAG, confirming anti-FLAG as a clean antibody for the activity-based protein profiling analysis. Reacting HEK293T cell lysates with all four probes led to the formation of a slew of enzyme-ubiquitin conjugates that were strongly detected by anti-FLAG. The four labeled samples showed clearly multiple similarly positioning bands in the Western blot that had likely resulted from same cysteine enzymes. However, there are bands that were uniquely present in the ubiquitin azapeptide ester-labeled samples but weakly or not detectable in the FlagUbPa-labeled sample. For example, there was an exceptionally strong Western blot band at 37 kDa for all three azapeptide ester probe-treated samples. Although the FlagUbPa-treated sample has a band at a similar position, it is significantly weaker and difficult to assess whether this is from another enzyme. A negative control was also conducted with Flag-Ub_1‑75_-Cys-6×His. Incubating HEK293T cell lysates with Flag-Ub_1‑75_-Cys-6×His did not result in any significant labeling of proteins as shown in Figure S15B, confirming that chemical reactivity at the *C*-terminus of FlagUbPa and three ubiquitin azapeptide ester probes toward an activated cysteine in an enzyme is required for profiling cysteine enzymes in the ubiquitination/deubiquitination cascade.

**Figure 5:**
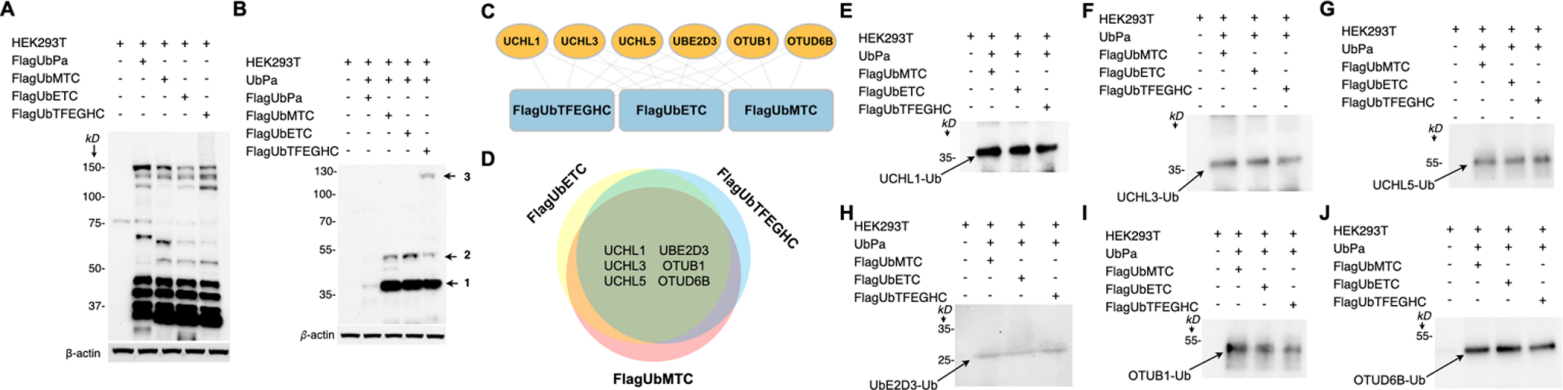
Activity-based protein profiling using three ubiquitin azapeptide ester probes. (A) Cysteine enzymes profiled from HEK293T cell lysates using FlagUbPa and three ubiquitin azapeptide ester probes. Cell lysates were reacted with four probes for 20 h before their SDS-PAGE analysis and Western blotting by anti-FLAG. (B) Cysteine enzymes from HEK293T cells profiled specifically by three ubiquitin azapeptide ester probes. Cell lysates were first reacted with UbPa for 12 h to remove UbPa-reacting enzymes and then treated with FlagUbPa and three ubiquitin azapeptide ester probes for an additional 12 h before their SDS-PAGE analysis and Western blotting by anti-FLAG. Enzymes that were uniquely labeled by ubiquitin azapeptide ester probes are indicated. (C) Cross-interaction network of identified cysteine enzymes from samples in B. (D) Venn diagram illustrated the distribution of same enzymes presented in C. (E–J) Western blot validation of UCHL1, UCHL3, UCHL5, UbE2D3, OTUB1, and OTUD6B in anti-FLAG immunoprecipitated samples from HEK293T cell lysates treated with UbPa for 12 h and then three ubiquitin azapeptide esters for additional 12 h. Final Western blot detection was completed using corresponding antibodies for 6 named enzymes.

To further explore cysteine enzymes that may be uniquely labeled with ubiquitin azapeptide esters, we profiled cysteine enzymes with three ubiquitin azapeptide ester probes from HEK293T cell lysates that were pretreated first with UbPa to inactivate UbPa-reactive enzymes. UbPa lacks a FLAG tag to avoid Western blot detection by anti-FLAG. To synthesize UbPa, we expressed and purified Ub_1‑75_-Cys-6×His (Figures S2A,B and S16) and used it to synthesize UbPa by incubation with TCEP, NTCB, and propargylamine (Figure S17A). Successfully obtained UbPa was confirmed by ESI-MS (Figure S17B). We used UbPa to treat HEK293T cell lysates for 12 h at 4 °C first. UbPa-treated HEK293T cell lysates were then reacted separately with FlagUbPa and three ubiquitin azapeptide ester probes for additional 12 h. All samples were then analyzed by SDS-PAGE and blotted by anti-FLAG (Figure 5B). The sample pretreated with UbPa, followed by FlagUbPa, showed only a faint band near 37 kDa, indicating that almost all DUBs capable of reacting with a propargylamine-conjugated ubiquitin probe were inactivated by UbPa. In contrast, the three UbPa-treated samples that were further incubated with ubiquitin azapeptide ester probes displayed distinct bands near 37 kDa (band **1**) and 50 kDa (band **2**). Additionally, in the FlagUbTFEGHC sample, a unique band around 130 kDa (band **3**) was detected. Several other weak bands were also visible in the azapeptide peptide ester-treated samples. The strong intensity of band **1** and the presence of additional bands in the azapeptide ester-treated samples approve that UbPa has low reactivity toward certain enzymes. The successful covalent labeling of these enzymes by the ubiquitin azapeptide ester probes supports the idea that Ub azapeptide ester probes display superior reactivity toward certain cystine enzymes compared to FlagUbPa. Consequently, their application in activity-based protein profiling may help identify enzymes that are unresponsive to FlagUbPa.

### Proteomic Characterization of Cysteine Enzymes Uniquely Profiled by Ubiquitin Azapeptide Esters

To characterize cysteine enzymes uniquely labeled by the three ubiquitin azapeptide ester probes in UbPa-pretreated HEK293T cell lysates, we immunoprecipitated probe-conjugated proteins using anti-FLAG magnetic agarose beads. The immunoprecipitated proteins were subjected to trypsin digestion, followed by liquid chromatography-tandem mass spectrometry (LC-MS/MS) analysis for peptide sequencing and protein identification. The identified proteins are listed in Table S3, including five deubiquitinating enzymes (DUBs): UCHL1, UCHL3, UCHL5, OTUB1, and OTUD6B. Interestingly, one E2 enzyme, UbE2D3, was also identified and labeled by all three azapeptide ester probes, confirming that ubiquitin azapeptide ester probes can profile non-DUB cysteine enzymes in the ubiquitination/deubiquitination cascade as well. A cross-interaction network and a Venn diagram depicting the relationship of these enzymes and their labeling by the three probes are shown in Figure 5C,D, respectively. To validate the identification of all six enzymes, we performed SDS-PAGE analysis of anti-FLAG immunoprecipitated samples, followed by western blotting using antibodies specific to UCHL1, UCHL3, UCHL5, OTUB1, OTUD6B, and UbE2D3. As shown in Figure 5E–J, clear enrichment of bands corresponding to complexes formed between these enzymes and ubiquitin azapeptide ester probes was observed. Similar bands were absent in the HEK293T cell lysate control sample, underscoring the specificity and efficiency of the azapeptide ester probes in profiling distinct cysteine enzymes involved in the ubiquitin signaling cascade.

Three DUBs including Ataxin-3, OTULIN, and OTUD7C that exhibited low reactivity toward FlagUbPa but were efficiently labeled by ubiquitin azapeptide ester probes in the biochemical assays shown in Figure 3 were not identified in the proteomic analysis. This discrepancy may be attributed to their low abundance in HEK293T cells or limitations inherent in the proteomic analysis setup. To directly assess Ataxin-3 labeling by all four probes in HEK293T cell lysates, we incubated the lysates with each probe separately for 20 h, followed by SDS-PAGE and Western blotting using an anti-Ataxin-3 antibody. As shown in Figure S19, FlagUbPa failed to label Ataxin-3, whereas all three ubiquitin azapeptide ester probes successfully formed covalent complexes with Ataxin-3, highlighting enhanced reactivity and target engagement of azapeptide ester probes compared to FlagUbPa for Ataxin-3 in a cellular environment. Ataxin-3 is a highly specialized DUB that preferentially cleave a polyubiquitin chain with more than 4 ubiquitin monomers.^40^ It also shows high isopeptide linkage selectivity during its catalyzed reactions. Ubiquitin azapeptide ester probes show better reactivity toward ataxin-3 than FlagUbPa is likely due to their better chemical reactivity and stronger binding to the enzyme.

### Kinetic Characterizations of Five Identified DUB Enzymes on Their Reactions with FlagUbPa and Three Ubiquitin Azapeptide Ester Probes

Five DUB enzymes identified in the proteomic analysis, including UCHL1, UCHL5, UCHL3, OTUB1, and OTUD6B, were used to conduct kinetic characterizations of their reactions with FlagUbPa and three ubiquitin azapeptide ester probes. The assay involved incubating a DUB enzyme with a probe for a total reaction time of 40 min at 37 °C and quenching the reaction by adding SDS-PAGE loading dye at various reaction time intervals including 0* (operation dead time <30 s), 5, 10, 20, and 40 min. The quenched reaction samples were then analyzed by SDS-PAGE and blotted by anti-FLAG. Results are presented in Figure 6. BSA was used as a loading control for this experiment (Figure S17). Compared to FlagUbPa that showed weak labeling with UCHL1 and had close to nondetectable labeling at and before 10 min, all ubiquitin azapeptide ester probes reacted with UCHL1 strongly and displayed clearly visible labeling of UCHL1 at 0* min at which time a DUB enzyme was quickly mixed with a probe and the SDS-PAGE loading dye was added immediately after that (Figure 6A). Saturation labeling was observed starting from 5 min for all three ubiquitin azapeptide esters. For FlagUbTFEGHC, strong labeling was detected at 0* min indicating a very rapid reaction between UCHL1 and FlagUbTFEGHC. In comparison, even at 40 min, FlagUbPa displayed labeling of UCHL1 that is clearly not at the saturation level. Like UCHL1, UCHL5 reacted slowly with FlagUbPa and showed weak labeling even at 40 min (Figure 6B). However, it displayed labeling with all three ubiquitin azapeptide esters even at 0* min. Starting from 5 min, UCHL5 labeling with FlagUbMTC and FlagUbETC reached saturation. Surprisingly, UCHL5 labeling with FlagUbTFEGHC reached saturation at 0* min, indicating superior reactivity. For UCHL3, its labeling with all four probes reached saturation at 0* min (Figure 6C). Given its high reactivity with FlagUbPa, we suspect that its identification in the proteomic analysis might be due to its high abundance. For OTUB1, its labeling with FlagUbMTC outperformed the other three probes (Figure 6D). It exhibited strong labeling with FlagUbMTC at 0* min and the labeling reached saturation at 5 min. For the other three probes, their labeling of OTUB1 continued to rise from 0 to 40 min. For OTUD6B, three ubiquitin azapeptide esters outperformed FlagUbPa in its labeling, although for all probes the labeling continued to rise from 0 to 40 min (Figure 6E). Overall, kinetic labeling results align with the proteomic findings, though it is important to note that binding partners in cell lysates may have significant impacts on the proteomic results.

**Figure 6:**
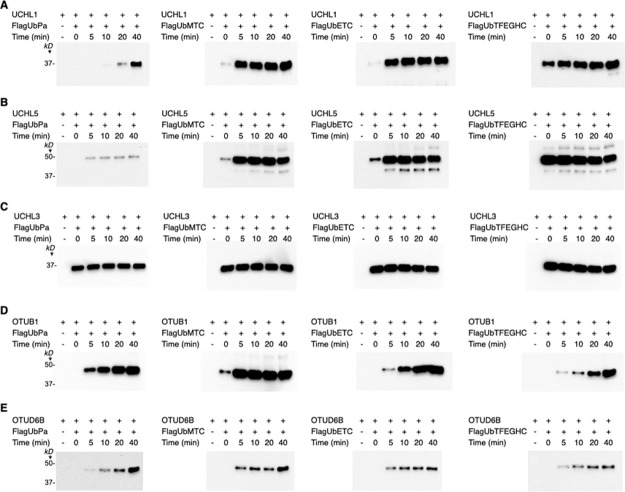
Covalent labeling trajectories of 5 DUBs including (A) UCHL1, (B) UCHL5, (C) UCHL3, (D) OTUB1, and (E) OTUD6B with four synthetic ubiquitin probes analyzed by anti-Flag-based Western blot analyses. UCHL1 and UCHL5 showed far quicker reaction kinetics with three ubiquitin azapeptide ester probes than with FlagUbPa.

### Inhibitory Effects of FlagUbPa and Ubiquitin Azapeptide Ester Probes on the Enzymatic Activities of UCHL1, UCHL5, and USP25

Figure 6 demonstrates that ubiquitin azapeptide ester probes exhibit significantly stronger labeling of UCHL1 and UCHL5 compared to FlagUbPa. To further investigate this observation, we synthesized ubiquitin-7-amino-4-coumarinyl-acetic acid (UbACA), as shown in Figure 7A, as a fluorogenic substrate for DUBs. The ACPL reaction was carried out between Ub1-75-Cys-6×His and Gly-ACA to make UbACA. We chose Gly-ACA over Gly-AMC (7-amino-4-methylcoumarin) due to its superior water solubility. The successful synthesis of UbACA was confirmed by ESI-MS, which showed a molecular weight consistent with its theoretical value (Figure S22). UbACA serves as a potential fluorogenic substrate for DUBs, where its catalytic hydrolysis by a DUB releases ACA, a highly fluorescent compound that can be detected using a plate reader. To validate UbACA as a substrate for UCHL1 and UCHL5, we reacted 2 μM UbACA with 20 nM UCHL1 or UCHL5 and measured time-dependent fluorescence changes (Excitation: 360 nm, Emission: 460 nm). The results, shown in Figure 7B, confirm that both UCHL1 and UCHL5 are active toward UbACA. USP25 was also included in this analysis as a candidate, given its unequal labeling efficiency with the four synthesized FLAG-containing ubiquitin probes as shown in Figure 3A vii. As shown in Figure 7B, USP25 is also active toward UbACA. Using UbACA as a substrate, we proceeded to characterize the inhibitory effects of FlagUbPa and three ubiquitin azapeptide ester probes on UCHL1, UCHL5, and USP25. In these assays, 20 nM of each enzyme catalyzed ACA release from 2 μM UbACA in the presence of varying concentrations of the four synthetic probes. The initial ACA release velocities were plotted against probe concentrations, with results presented in Figure 7C–E. For both UCHL1 and UCHL5, FlagUbPa exhibited very weak inhibition, even at 2 μM, whereas FlagUbTFEGHC achieved nearly 100% inhibition at 300 nM. Both FlagUbMTC and FlagUbETC demonstrated strong inhibition of UCHL1, with each achieving 50% inhibition at 100 nM. Although these two probes showed weaker inhibition of UCHL5 compared to UCHL1, they still achieved approximately 50% inhibition of UCHL5 at 2 μM. For USP25, FlagUbMTC exhibited the highest inhibition potency, followed by FlagUbTFEGHC, FlagUbETC, and finally FlagUbPa, although the differences in inhibition potency among the four probes were less pronounced compared to UCHL1 and UCHL5.

**Figure 7:**
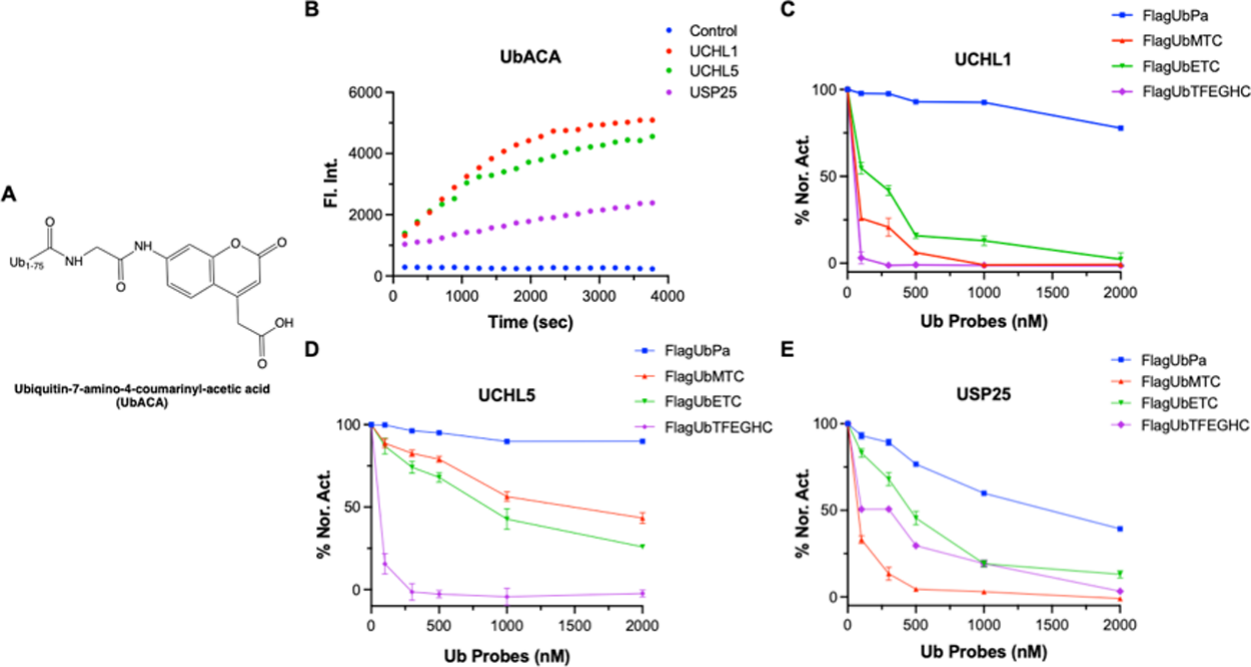
Characterization of four synthetic ubiquitin probes on their inhibition of DUB-catalyzed fluorescent 7-amino-4-coumarinyl-acetic acid (ACA) release from fluorogenic UbACA. (A) Structure of UbACA. (B) Catalytic release of ACA from UbACA by UCHL1, UCHL5, and USP25. 2 μM UbACA was incubated with 20 nM DUB 20 nM, and fluorescence increase (Ex: 360 nM; Em: 460) was then monitored. (C–E) Inhibition curves of UCHL1, UCHL5, and USP25 by four synthetic ubiquitin probes characterized by enzyme activity on UbACA. 20 nM DUB was used to catalyze ACA release from 2 μM UbACA in the presence of various concentrations of a ubiquitin probe. The initial 5 min reaction velocities for ACA release were used to plot inhibition curves.

While these kinetic assays were conducted under competitive inhibition conditions, we did not directly determine *K*_i_ values for each probe, as all four probes covalently react with the enzymes. Nonetheless, the results strongly support the conclusion that the ubiquitin azapeptide ester probes act as better mimics of native ubiquitinated protein substrates and exhibit superior interaction with UCHL1, UCHL5, and USP25 compared to FlagUbPa. Additionally, these findings are consistent with the proteomic results.

### Activity-Based Labeling of Cysteine Enzymes in the Ubiquitination/Deubiquitination Cascade from Mouse Tissues Using Ubiquitin Azapeptide Ester Probes

Encouraged by protein profiling results from HEK293T cell lysates, we proceeded to test three ubiquitin azapeptide esters as activity-based probes to label cysteine enzymes in the ubiquitination/deubiquitination cascade from mouse tissues. FlagUbPa was used as a control as well. Mouse tissues including kidney, brain, liver, and lung were dissected and immediately stored on dry ice. Homogenized tissues from 20–50 mg tissue samples were clarified and supernatants were then used to react separately with all four probes for 20 h at 4 °C. Final reaction mixtures were then separated by SDS-PAGE and blotted by anti-FLAG. Results are presented in Figure 8A–D. To probe cysteine enzymes that may be uniquely modified by ubiquitin azapeptide esters but not by FlagUbPa, additional labeling experiments were conducted where all mouse tissue lysates were incubated with UbPa for 12 h before a ubiquitin azapeptide ester was added for an additional incubation period of 12 h. Final reaction samples were then separated by SDS-PAGE and blotted by anti-FLAG. Results from these experiments are presented in Figure 8E–H. Figure 8A–D revealed distinct cysteine enzyme labeling patterns by all four probes across different tissues, indicating different proteomes of cysteine enzymes in the ubiquitination/deubiquitination cascade are expressed in these tissues. Interestingly, ubiquitin azapeptide esters consistently provided increased labeling of cysteine enzymes compared to FlagUbPa in all mouse tissue lysates. Remarkably, at least one ubiquitin azapeptide ester consistently demonstrated superior labeling efficiency compared to FlagUbPa, suggesting enhanced substrate engagement and reactivity. We strongly believe that these results are attributed to the feature that ubiquitin azapeptide esters are better than other conventional activity-based probes in mimicking ubiquitinated proteins when reacting with cysteine enzymes. It is also notable that kidney and liver exhibited higher levels of labeled cysteine enzymes compared to brain and lung. These different labeling patterns may be attributed to diverse metabolic activities and/or cellular processes inherent to each tissue type. Kidney and liver, known for their involvement in detoxification and complex metabolic pathways, require a multitude of complex enzymes. The increased abundance of cysteine enzymes in the ubiquitination/deubiquitination cascade in the two tissues likely contributes to enhanced labeling with all four probes. In contrast, lower levels of labeling observed for brain and lung suggest a reduced abundance of cysteine enzymes in the ubiquitination/deubiquitination cascade involved in metabolic and/or other cellular processes. Figure 8E–H reveal cysteine proteases that cannot be fully removed by UbPa. As a matter of fact, all four tissue lysates display cysteine enzymes intensely labeled with ubiquitin azapeptide esters after their treatment with UbPa. And there are a significant number of intensely labeled bands for kidney, lung, and liver. Although brain tissue lysates showed fewer labeled bands, there are still several significantly labeled bands including the one around 37 kDa. Once again, labeling patterns in Figure 8E–H are distinct from each other indicating unique cysteine proteomes in four tissues. Comparatively, we can conclude that ubiquitin azapeptide esters serve as better activity-based probes than FlagUbPa to pull out more enzymes. However, due to different reactivities of all probes, we think it will work better if a combination of ubiquitin probes with different reactive *C*-terminal groups are used for thorough activity-based protein profiling of cysteine enzymes in the ubiquitination/deubiquitination cascade.

**Figure 8:**
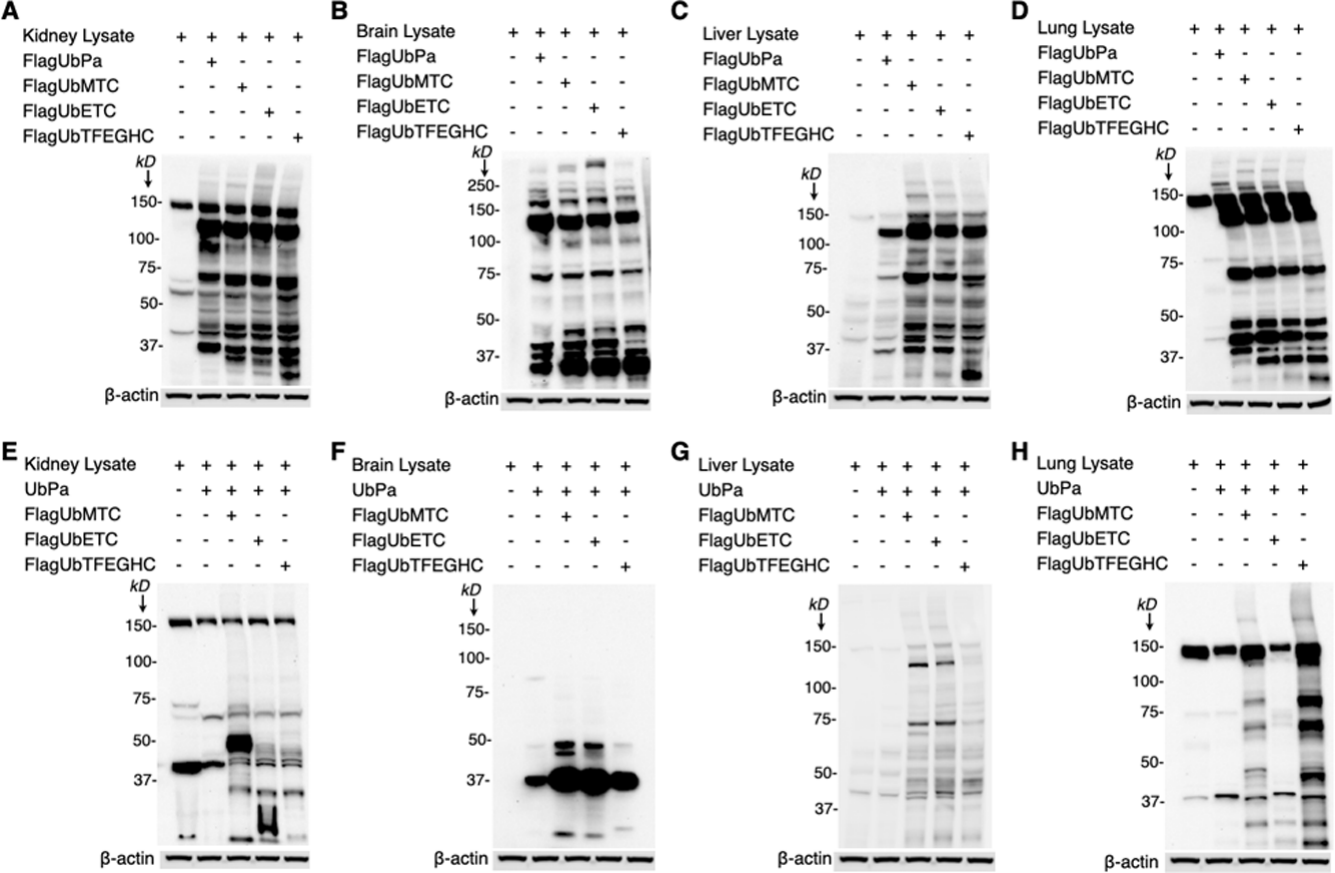
Profiling cysteine enzymes in the ubiquitination/deubiquitination cascade in different mouse tissues including kidney (A), brain (B), liver (C), and lung (D) by four synthetic ubiquitin probes and profiling the same tissues, kidney (E), brain (F), liver (G), and lung (H), with three ubiquitin azapeptide ester probes after their reaction with UbPa to remove UbPa-reacting enzymes. For all tissues, there are bands clearly visible for three ubiquitin azapeptide ester probes but not for FlagUbPa.

## Conclusions

Ever since the discovery of ubiquitin in 1975,^41^ the significance and diverse role of this small 76aa protein have been continuously expanding.^1,3,42–44^ Enzymes that regulate ubiquitination have been long known implicated in the development of various diseases.^45^ Some of these enzymes have been successfully targeted for disease intervention.^46^ Given the large number of known enzymes that are involved in the ubiquitination/deubiquitination cascade, collective studies of them are important in understanding the overall ubiquitin biology and disease progressions. A great tool of this type of studies are activity-based ubiquitin probes that allow proteomic analysis of cysteine enzymes in this signal pathway. Novel enzymes in this pathway has also been continuously increasing. Their identification may also be facilitated by activity-based ubiquitin probes. There are a number of activity-based ubiquitin probes that have been developed based on different chemistry mechanisms. A tribute needs to be paid to late Huib Ovaa who was a pioneer in this research field and contributed to the development of the majority of these probes.^47^ However, most of these probes are structurally different from a ubiquitinated protein substrate and engage enzymes in the ubiquitination/deubiquitination cascade using chemical mechanisms different from the enzyme catalysis. We designed ubiquitin azapeptide esters as alternatives of conventional activity-based ubiquitin probes. Based on chemistry mechanisms of ubiquitin azapeptide esters in engaging cysteine enzymes, we suspected that they may serve as general covalent probes for cysteine enzymes in the ubiquitination/deubiquitination cascade. As expected, our results confirmed this aspect. Activity-based protein profiling of cysteine enzymes in the ubiquitination/deubiquitination cascade in HEK293T cell lysates and mouse tissue lysates also approved that ubiquitin azapeptide esters are better than a commonly used UbPa probe in labeling cysteine enzymes in both HEK293T cell lysates and mouse tissue lysates. Given all the results we obtained, we can conclude that ubiquitin azapeptide esters serve as novel and optimal activity-based probes for mapping activities of cysteine enzymes in the ubiquitination/deubiquitination cascade.

One expected observation that was made during our research was different cysteine enzyme labeling patterns for the four mouse tissue lysates. This reflects different roles that cysteine enzymes in the ubiquitin signal pathway play in these tissues. This observation underscores the importance of considering tissue-specific factors in studying the ubiquitination/deubiquitination cascade. It is also interesting to observe that different probes can also lead to different labeling results. Although we believe ubiquitin azapeptide esters serve as optimal activity-based ubiquitin probes for cysteine enzymes in the ubiquitination/deubiquitination cascade, we think it will be generally a good idea to use a combination of several probes that work with different chemistry mechanisms to conduct activity-based protein profiling in order to thoroughly pull down these enzymes. Given all the differences observed for the four tissue lysates, our next goal is to identify active, tissue-specific enzymes using our developed ubiquitin azapeptide ester probes. Another interesting aspect is whether these probes can be used to study different disease cells to identify enzymes whose activities are disrupted. These studies may potentially lead to the identification of novel therapeutic targets.

In conclusion, we designed ubiquitin azapeptide esters as novel activity-based ubiquitin probes and demonstrated that they are generally better activity-based probes for DUBs than FlagUbPa that has a propargylamine reactive group. Comparative studies demonstrated that ubiquitin azapeptide esters serve as optimal next-generation probes for profiling cysteine enzymes in the ubiquitination/deubiquitination cascade in both cell and mouse tissue lysates. As next-generation activity-based probes, we believe that ubiquitin azapeptide esters may be broadly applied to the characterization of cysteine enzymes in the ubiquitination/deubiquitination cascade. Applying these probes to disease models (e.g., cancer, neurodegeneration) could uncover dysregulated DUB activity, leading to the identification of novel therapeutic targets, which will be our future research direction.

## Supplementary Material



## Notes

Experimental procedures for protein expression, ubiquitin probe synthesis, characterization of probes and their reactions with different enzymes, protein profiling using synthesized probes from cell and tissue lysates, validation of identified enzymes from proteomic analysis, kinetic characterization of identified DUBs, UbACA synthesis and its use in characterization of different probes in inhibiting DUBs, and synthesis and use of biotin-GG-LRG-methyl carbazate, as well as supplementary figures and tables (PDF)
