# Supplementary material for: Ubiquitin Azapeptide Esters as Next-Generation Activity-Based Probes for Cysteine Enzymes in the Ubiquitin Signal Pathway
Source: J Am Chem Soc. 2025 Apr 23;147(21):17817–28. doi: 10.1021/jacs.5c01732 (PMC12119208; doi:10.1021/jacs.5c01732)
Supplement: Supplementary file 1 [file ja5c01732_si_001.pdf]

Supplementary Information for

Ubiquitin Azapeptide Esters as Next-Generation Activity-Based Probes for Cysteine Enzymes in the Ubiquitin Signal Pathway

Saibal Chanda[a], Sandeep Atla[b], Xinlei Sheng[c], Satyanarayana Nyalata[b], Yugendar R. Alugubelli[b], Demonta D. Coleman[b], Wen Jiang[d], Rosana Lopes[a], Shaodong Guo[d], A. Joshua Wand[a,b,f], Yingming Zhao[c], and Wenshe Ray Liu[a,b,e,f,*]

[a]Department of Biochemistry and Biophysics, College of Agriculture and Life Sciences, Texas A&M University, College Station, TX 77843

[b]Texas A&M Drug Discovery Center and Department of Chemistry, College of Arts and Sciences, Texas A&M University, College Station, TX 77843

[c]Ben May Department of Cancer Research, The University of Chicago, IL 60637

[d]Department of Nutrition, College of Agriculture and Life Sciences, Texas A&M University, College Station, TX 77843

[e]Institute of Biosciences and Technology and Department of Translational Medical Sciences, School of Medicine, Texas A&M University, Houston, TX 77030

[f]Department of Cell Biology and Genetics, School of Medicine, Texas A&M University, College Station, TX 77843

*Correspondence should be addressed to Wenshe Ray Liu: wsliu2007@tamu.edu

Materials and Methods

Compounds. Methyl hydrazinecarboxylate (MTC) and Ethyl hydrazinecarboxylate (ETC), TCEP and NTCB were purchased from A2B chem LLC, San Diego, CA, USA. Propargylamine was purchased from TCI Chemicals, America.

Expression and Purification of All Ubiquitin Proteins. All ubiquitin proteins including Ub_{1-75}-Cys-6×His, Flag-Ub_{1-74}-Cys-6×His, and Flag-Ub_{1-75}-Cys-6×His were recombinantly expressed and purified. An overnight culture of *E. coli* BL21(DE3) cells harboring a pETDuet-1 expression vector coding Ub_{1-75}-Cys-6×His, Flag-Ub_{1-74}-Cys-6×His, or Flag-Ub_{1-75}-Cys-6×His was inoculated (1:100 dilution) into a 2xYT medium containing 100 µg/mL ampicillin. Cells were let grow at 37 ºC until OD_{600} reached 0.6-0.9 and 1 mM IPTG was added to induce protein expression. The cells were let grow in a shaker at 37 ºC for 8 h or 18 ºC for 16 h and collected by centrifugation (5,000 rpm, 30 min, 4 ºC). The cell pellets were resuspended in a 100 mL lysis buffer (50 mM NaH_2PO_4, 500 mM NaCl, 5 mM imidazole, 1 mM TCEP, pH 7.8) and lysed by sonication. The cell lysates were clarified by centrifugation (10,000 rpm, 30 min, 4 ºC) and the supernatant was decanted. 6 M HCl solution was then added dropwise into the supernatant to adjust pH to 2 to drive the formation of white precipitate inside the solution.The precipitate was pelleted by centrifugation (10,000 rpm, 30 min, 4 ºC) and removed. The pH value of the supernatant was then adjusted back to 7.8 by adding 6 M NaOH. This supernatant was loaded onto high-affinity Ni^{2+}-NTA resins at RT and then washed with a buffer (50 mM NaH_2PO_4, 500 mM NaCl, 30 mM imidazole, 1 mM TCEP, pH 7.8). The bound protein was eluted by another buffer (50 mM NaH_2PO_4, 250 mM NaCl, 300 mM imidazole, 1 mM TCEP, pH 7.8) and then desalted into a 50 mM ammonium bicarbonate (ABC) buffer using HiPrep 26/10 Desalting column (Cytiva). All proteins were concentrated using 3 kDa MWCO Amicon ultracentrifugation filter devices (Millipore Sigma) and concentrations were measured using a NanoDrop spectrophotometer (ThermoFisher). Eventually, all proteins were aliquoted, lyophilized, and stored at -80 ºC.

Use of ACPL to Synthesize Ubiquitin Azapeptide Ester Probes. A 500 mM TCEP stock solution was prepared in water, and a 500 mM NTCB stock solution in DMSO. Solutions of Propargylamine (Pa), Methyl hydrazinecarboxylate (MTC), and Ethyl hydrazinecarboxylate

(ETC) were made in water at 2 M each, while a 2 M solution of 2,2,2-trifluoroethyl-2-glycylhydrazine-1-carboxylate (TFEGHC) was prepared in DMSO.

Synthesis of FlagUbMTC and FlagUbETC. To initiate the reaction, Flag-Ub$_{1-75}$-Cys-6×His protein pellets were dissolved in MTC and ETC solutions. TCEP and NTCB were added to a final concentration of 5 mM each, and the pH was adjusted to 8. The mixtures were incubated at 37 °C. desalted using a HiTrap column, and then incubated with Ni^{2+}-NTA resins and were removed with micro syringe filters, and the flowthroughs were collected for ESI-MS analysis.

Synthesis of FlagUbTFEGHC. Flag-Ub$_{1-74}$-Cys-6×His protein pellets were dissolved in water, followed by the addition of TFEGHC, TCEP (5 mM), and NTCB (5 mM). The pH was adjusted to 8 and the mixture was incubated at 37 °C. The reaction was desalted using a HiTrap column, then incubated with Ni^{2+}-NTA resins and were removed with micro syringe filters, and the flowthroughs were collected for ESI-MS analysis.

Synthesis of FlagUbPa and UbPa. Flag-Ub$_{1-75}$-Cys-6×His and Ub$_{1-75}$-Cys-6×His protein pellets were dissolved in 500 mM propargylamine, with TCEP and NTCB added (5 mM each). The pH was adjusted to 9 and the mixtures were incubated at 37 °C. After desalting with a HiTrap column, the products were incubated with Ni^{2+}-NTA resins and were removed with micro syringe filters, and the flowthroughs were collected for ESI-MS analysis.

Western Blot Analysis for FlagUbPa and Ubiquitin Azapeptide Ester Probes. To assess the functionality of the Flag tag linked to the synthesized Ubiquitin probes, all probes underwent SDS-PAGE (mPAGE 4-12% bis-tris gel, Millipore Sigma) followed by western blotting. A peroxidase (HRP)-conjugated anti-FLAG antibody (Millipore Sigma), capable of binding the Flag tag, was utilized for detection.

Covalent Capture of USP5 by Ubiquitin Azapeptide Ester Probes. 0.1µg/µL USP5 (Sino Biological) and 0.1µg/µL all ubiquitin activity-based probes were mixed, and the final reaction volume was adjusted into a 20 µL DUB reaction buffer (50 mM HEPES, 100 mM NaCl, 5 mM TCEP pH 7.85). All reactions were incubated at 37 ºC for 40 min and quenched by adding a 6×

SDS loading dye containing 30% β-mercaptoethanol. The reaction mixtures were then analyzed by SDS-PAGE (4-12% bis-tris gel) and Western blotting using HRP-conjugated anti-FLAG.

Covalent complex formation between FlagUbPa, Ub azapeptide probes, and DUBs. 0.1µg/µL USP2 CD (R&D systems), USP7 CD (Medchem express), USP8 (Boston biochem), USP10 (R&D systems), USP14 (UBP bio), GST-USP15 (UBP bio), USP25 (R&D systems), CYLD (Sino biological), UCHL3 (UBP bio), GST-Ataxin3 (UBP bio), GST-JOSD2 (UBP bio), OTUB2 (UBP bio), OTULIN (UBP bio), Cezanne-OTUD7B (UBP bio), OTUD2 (UBP bio), OTUD7C (UBP bio) and 0.1µg/µL all ubiquitin probes were mixed, and the final reaction volume was adjusted into a 20 µL DUB reaction buffer (50 mM HEPES, 100 mM NaCl, 5 mM TCEP pH 7.85). All reactions were incubated at 37 ºC for 40 min and quenched by adding a 6× SDS loading dye containing 30% β-mercaptoethanol. The reaction mixtures were then analyzed by SDS-PAGE (4-12% bis-tris gel) and Western blotting using HRP-conjugated anti-FLAG.

The reaction of all DUBs with biotin-GG-LRG-MTC peptide: 0.1µg/µL of each of the DUBs (mentioned above) and the peptide were mixed, and the final reaction volume was adjusted into a 20 µL DUB reaction buffer (50 mM HEPES, 100 mM NaCl, 5 mM TCEP pH 7.85). All reactions were incubated at 37 ºC for 40 min and quenched by adding a 6× SDS loading dye containing 30% β-mercaptoethanol. 2µM streptavidin was added to each reaction mixture followed by SDS-PAGE (4-12% bis-tris gel) and Western blotting using anti-streptavidin antibody (R&D systems) and detected using an anti-rabbit secondary antibody (Abcam).

Labeling of E1 Activating Enzyme. E1 labeling experiments were performed in a ubiquitination buffer containing 50 mM HEPES, 100 mM NaCl, 5 mM TCEP (pH 7.85), 0.1 µg/µL UBE1 (UBP bio) and ubiquitin probes. The reaction mixtures were incubated at 37 ºC. Reactions were quenched by adding a 6× SDS loading dye containing 30% β-mercaptoethanol. The mixtures were then analyzed by SDS-PAGE (4-12% bis-tris gel) and Western blotting using HRP-conjugated anti-FLAG.

Labeling of E2 Conjugating Enzyme. E2 labeling experiments were performed in a ubiquitination buffer containing 50 mM HEPES, 100 mM NaCl, 5 mM TCEP (pH-7.85), 0.1 µg/µL UBE2E2 (UBP bio), and ubiquitin probes. The reaction mixtures were incubated at 37 ºC. Reactions were quenched by adding a 6× SDS loading dye containing 30% β-mercaptoethanol. The mixtures were then analyzed by SDS-PAGE (8-16% bis-tris gel) and Western blotting. The blot was probed with an anti-UBE2E2 primary antibody (Invitrogen) derived from rabbit and detected using an HRP-conjugated goat anti-rabbit secondary antibody (Abcam).

Labeling of Parkin. Parkin was recombinantly expressed in *E. coli*. Labeling of Parkin was performed in a ubiquitination buffer containing 50 mM HEPES, 100 mM NaCl, 5 mM TCEP (pH-7.85), 0.1 µg/µL Parkin, and ubiquitin probes. The reaction mixtures were incubated at 37 ºC. Reactions were quenched by adding a 6×SDS loading dye containing 30% β-mercaptoethanol. The mixtures were then analyzed by SDS-PAGE (4-12% bis-tris gel) and Western blotting using HRP-conjugated anti-FLAG.

***In-vitro* labeling of UBE3A and ITCH.** Labeling experiment of UBE3A was performed in a ubiquitination buffer containing 50 mM HEPES, 100 mM NaCl, 5 mM TCEP (pH 7.85), 0.1 µg/µL UBE3A (R&D Systems), ITCH (UBP bio) and the ubiquitin probes. The reaction mixtures were incubated at 37 ºC before they were quenched by adding a 6×SDS loading dye containing 30% β-mercaptoethanol. The mixtures were then analyzed by SDS-PAGE (4-12% bis-tris gel) and Western blotting using HRP-conjugated anti-FLAG.

Profiling of Active Ubiquitination/Deubiquitination Cysteine Enzymes in the HEK293T cell lysate using Ubiquitin Probes. HEK293T cells were cultured in DMEM (Gibco) supplemented with 10% fetal bovine serum (FEB, Gibco) and 5% penicillin-streptomycin (Pen-Strep, Sigma; Pen: 10,000 units/Strep: 10 mg/mL). The cells were grown in T75 flasks in a 37 ºC, 5% CO_2 incubator until they reached approximately 90% confluency. Subsequently, the cells were collected in 1× PBS buffer and transferred into 1.5 mL tubes. To lyse the cells, Pierce (ThermoFisher) IP protease-free cell lysis buffer was added to the cell suspension. The mixture was gently shaken under refrigeration for 30 min to ensure cell lysis. After the cell lysis process,

the cell lysate was clarified by centrifugation at 14,000 rpm for 30 min at 4 ºC. The resulting supernatant was collected, and the protein concentration was measured by the BCA assay.

Profiling Using Four Flag-Fused Ubiquitin Activity-based Probes. HEK293T cell lysates were incubated separately with one of four probes for 20 h at 4 ºC before they were analyzed using a 4-12% bis-tris gel and subjected to Western blotting using HRP-conjugated anti-FLAG.

Profiling After depleting UbPa-binding Cysteine Enzymes. In a separate set of profiling experiments, four aliquoted HEK293T cell lysates were initially incubated with UbPa for 12 h at 4 ºC. Afterward, three Flag-fused ubiquitin azapeptide ester probes were added separately to three different reaction mixtures, and the incubation continued for another 12 h at 4 ºC. Subsequently, all reactions were analyzed using a 4-12% bis-tris gel followed by Western blotting with HRP-conjugated anti-FLAG.

Immunoprecipitation. Anti-DYKDDDDK magnetic agarose beads (Pierce, Thermo Scientific) were equilibrated to room temperature and thoroughly mixed. A 50 µL slurry of beads was transferred to a microcentrifuge tube and washed twice with binding buffer (25 mM Tris-HCl pH 7.4, 150 mM NaCl, 1 mM EDTA, 1% NP-40, 5% glycerol). Probe-treated cell lysates were added and incubated at room temperature with mixing. The beads were then washed three times with 1× PBS and once with purified water. Bound proteins were acid-eluted and pH was adjusted with neutralization Buffer. The elution was repeated three times for maximum recovery.

Proteomic Identification of Ubiquitination/Deubiquitination Cysteine Enzymes in HEK293T Cell Lysates. *Sample preparation*. The eluted proteins from the previous immunoprecipitation procedure were reduced with 25 mM Dithiothreitol at 70°C for 20 min and alkylated with 50 mM iodoacetamide at room temperature for 30 min in the dark. To acidify samples, 12% phosphoric acid was added to achieve a final concentration of 1.2%. The acidified samples were applied to the S-Trap columns (Protifi, C02-micro) pre-loaded with 165 µl 100 mM TEAB (pH 7.1) in methanol, followed by 5 washes using 150 µl 100 mM TEAB pH 7.1 in methanol. After the last wash, the protein samples underwent on-column digestion with trypsin solution (2 µg trypsin in 25 mM TEAB pH 8.0) for 14 hrs at 37°C. The resulting peptides were subsequently eluted with 40 µl each

of 50 mM TEAB (pH 8.0), 0.2% formic acid, and 0.2% formic acid in 50% acetonitrile. The eluted peptides were dried using speed vac and reconstituted in 1% formic acid and 2% acetonitrile, and they were applied to mass spectrometry analysis.

MS data analysis. The peptide samples were analyzed by an Orbitrap Exploris™ 480 mass spectrometer (Thermo Fisher Scientific, Inc., Waltham, MA) coupled with an EASY-nLC 1000 system (Thermo Fisher Scientific, Inc., Waltham, MA). Peptides were separated by a home-made silica column (12 cm length × 3 μm ID) packed with C18 resin (Dr. Maisch GmbH, Beim Bruckle, Germany) using solvents A (0.1% formic acid in water) and B (0.1% formic acid in 97% acetonitrile), with a continuous gradient from 2 to 45% B over 90 min, at a flow rate of 200 ng/min. Full-scan mass spectra were surveyed from 300 to 1,100 using the Orbitrap mass analyzer with mass resolution of 60,000 under positive-ion mode. MS/MS was performed in a data-dependent mode, and the spectra were recorded in profile mode. The top 20 most intense ions were selected for MS/MS analysis at a resolution of 15,000 using higher energy collision-induced dissociation (HCD) with 30% normalized collision energy (NCE). Other relevant parameters used in the analysis: isolation window, 1.6 m/z; default charge, 2+; automatic gain control (AGC) target, standard; maximum IT, automatic; dynamic exclusion, automatic.

MS/MS spectra were analyzed by Proteome Discoverer (ver. 2.5, Thermo Fisher Scientific). Mass accuracy was recalibrated by the spectrum files RC node, and the Minora feature detector node was used to detect chromatographic peaks. All MS/MS spectra were searched against a reverse-concatenated human protein sequence database (UniProt-SwissProt, downloaded in March 2021) using SEQUEST HT node. Key criteria used for the SEQUEST HT data searching full trypsin enzyme specificity, maximum missed cleavages of 2, precursor mass tolerance of 10 ppm, fragment mass tolerance of 0.02 Da, static carbamidomethylation of cysteine, and dynamic modifications of methionine oxidation, protein N-terminal Met-loss and Met-loss + acetyl, and lysine acetylation. Spectra matches were evaluated by Percolator with a false discovery rate (FDR) cutoff of < 1% based on the reverse sequences of the selected UniProt-SwissProt database.

In the consensus workflow, the feature mapper node was selected for chromatographic retention time alignment between runs with maximum retention time window of 10 min with mass tolerance

of 5 ppm. Peptide abundances were normalized using Total Peptide Amount method. A minimum of 1 unique peptide was required for each protein group. Peptides containing dynamic modifications were excluded from protein quantification.

Validation of cysteine enzymes identified in proteomic analysis. The eluates from co-immunoprecipitation of UbPa-treated HEK293T cell lysates, followed by Flag-fused ubiquitin azapeptide probes, were analyzed using a 4-12% bis-tris gradient gel and Western blotting. The blot was probed with anti-UCHL1 (Invitrogen), anti-UCHL3 (Proteintech), anti-UCHL5 (Invitrogen), anti-UbE2D3 (Abcam) and anti-OTUD6B antibodies from rabbit, detected with an HRP-conjugated goat anti-rabbit secondary antibody (Abcam). To confirm the presence of OTUB1, anti-OTUB1 (Origene) from mouse was used, detected with an HRP-conjugated anti-mouse secondary antibody (R&D Systems).

Kinetic Characterization of DUBs with synthesized Ubiquitin Probes. A time dependent reaction between 5 DUBs: OTUB1 (Sino biological), UCHL1 (Sino Biological), UCHL3 (UBP bio), UCHL5 (UBP bio) and OTUD6B (UBP bio) and each of the four Flag-fused ubiquitin probes was carried out. Each reaction mixture comprised 0.1 µg/µL probes and DUBs, and 1 mM DTT in a 50 µL 1×PBS buffer. Additionally, control reactions, lacking the probe but containing the DUB, were prepared. All reactions underwent incubation at 37°C for various durations: 0, 5, 10, 20, and 40 min. Following each time interval, the reactions were stopped by adding a 6×SDS loading dye containing 30% β-mercaptoethanol. Subsequently, the samples were analyzed using 4-12% bis-tris gel and subjected to Western blotting with HRP-conjugated anti-FLAG.

Synthesis of UbACA using ACPL. Ub_{1-75}-Cys-6×His protein pellets were dissolved in 500 mM 2-(7-(2-aminoacetamido)-2-oxo-2H-chromen-4-yl) acetic acid with TCEP and NTCB added (5 mM each). The pH was adjusted to 8.5 with and the mixtures were incubated at 37 °C. After desalting with a HiTrap column, the products were incubated with Ni^{2+}-NTA resins and were removed with micro syringe filters, and the flowthroughs were collected for ESI-MS analysis. At this stage, hydrolyzed Ub and the probe UbACA were observed. UbACA was further purified by ion-exchange chromatography and the mass was confirmed by ESI-MS analysis.

Characterization of Inhibitory effects of Flag-fused probes on the enzymatic activity of DUBs. In a 96-well plate, 20 nM of each DUB, UCHL1 (Sino Biological), UCHL5 (UBP Bio), and USP25 (R&D Systems) was incubated separately with 2 µM UbACA. Following this, increasing concentrations of Flag-fused Ub probes (100 nM, 300 nM, 500 nM, 1 µM, and 2 µM) were added. Fluorescence measurements were taken at 30 °C for 1 hour (excitation at 360 nm and emission at 460 nm).

Labeling of Ataxin-3 by ubiquitin Probes in HEK293T Cell Lysates. HEK293T cell lysates were incubated separately with four Flag-fused ubiquitin activity-based probes for 20 h at 4 ºC. The samples were then analyzed by SDS-PAGE (4-12% bis-tris gel) and Western blotting. The blot was probed with an anti-ataxin-3 primary antibody (Invitrogen) derived from rabbit and detected using an HRP-conjugated goat anti-rabbit secondary antibody (Abcam).

Mapping Activities of Ubiquitination/Deubiquitination Cysteine Enzymes in Mouse tissue Lysates. Mouse tissues were dissected, followed by immediate placement on dry ice. After weighing the tissue samples, only 20-50 mg per sample was taken for preparing lysates. 20 µL of 1× RIPA buffer was added to per milligram of tissue (400-1000 µL total). Tissues were homogenized using a tissue homogenizer. The homogenized tissue lysates were centrifuged at 12,000 RPM at 4 °C for 15 min. The supernatant was decanted and transferred it into a clean tube. For the binding reactions, four different mouse tissue lysates (Liver, lungs, kidney, and brain) were incubated separately with each activity-based probe for 20 h at 4 ºC. The reaction was stopped by adding a 6× SDS loading dye containing β-mercaptoethanol. Finally, all reactions were analyzed using a 4-12% bis-tris gel and subjected to Western blotting using HRP-conjugated anti-FLAG.

In a separate set of binding reactions, four aliquoted mouse tissue lysates were initially incubated with UbPa for 12 h at 4 ºC. Afterward, three Flag-fused ubiquitin azapeptide ester probes were added separately to three different reaction mixtures, and the incubation continued for another 12 h at 4 ºC. The reaction was terminated by adding a 6×SDS loading dye containing 30% β-mercaptoethanol. Subsequently, all reactions were analyzed using SDS-PAGE (4-12% bis-tris gel) and subjected to Western blotting using HRP-conjugated anti-FLAG.

Biotin-GG-LRG-Methyl Carbazate Synthesis.

Glycine preloading to 2-chlorotrityl chloride resin. 2-chlorotrityl chloride resin (AAPPTEC #RTZ001) was swelled with 1.5 mL of degassed dichloromethane (DCM) for 5 min with gentle shaking. Fmoc-glycine (3 eq) and DIPEA (4 eq) were added to the swelled resin slurry and left to react for 5 min. Following the 5 min incubation, an additional 8 eq of DIPEA was added and the reaction was left for 1 h. After the coupling, the residual trityl groups on resin were encapped with the addition of methanol and mixing for 15 min. The resin was drained and washed with DCM (3 x 3 mL), DMF (3 x 3 mL), and methanol (3 x 3 mL). The glycine-loaded resin was then subjected to automated peptide synthesis.

Automated Peptide Synthesis. The remaining amino acids, Gly-Gly-Leu-Arg, were coupled to the glycine-loaded 2-chlorotrityl chloride resin using a MultiPep 2 Peptide Synthesizer (CEM). All amino acid derivatives were standard derivatives for Fmoc peptide synthesis commercially purchased from Chem-Impex. Fmoc-amino acids were deprotected using 20% piperidine in DMF (2 x 2 mL, 5 min at 50 °C). Amino acids were coupled using HATU/NMM double coupling cycles (4.2 eq amino acid, 4.2 eq HATU, 8 eq NMM, 15 min at 50 °C). Following synthesis, the resin was dried with ethanol washes (3 x 3 mL) followed by dichloromethane washes (3 x 3 mL) and evaporated under vacuum.

On-resin Peptide Biotinylation. Following the automated peptide synthesis, the resin was washed with DMF (3 x 3 mL), and 3 eq of Biotin-NHS (Chem Scene #CS-5714) in 1 mL of DMF was added. After, 6 eq of DIPEA was added to the mixture and the reaction was left overnight with gentle shaking. Biotinylation of the N-terminus was verified with a ninhydrin test.

Peptide Cleavage. The peptide was cleaved in 2 mL of 92.5:2.5.2.5:2.5 TFA:TIS:DODT:H2O for 1 h at room temperature. Following cleavage, the resin was filtered, and the peptide was precipitated from the filtrate by an 8-fold dilution in cold diethyl ether. After collection by centrifugation (5251 xg, 20 min), it was lyophilized to a powder overnight.

Methyl Carbazate Coupling. The peptide was dissolved in 1 mL of DMF before the addition of 6 eq methyl carbazate, 1.2 eq HATU, and 3 eq DIPEA. The reaction was left for 1.5 h at room

temperature. Following the reaction, the peptide solution was precipitated by an 8-fold dilution in cold diethyl ether. After centrifugation (5251 xg, 20 min), the peptide product was lyophilized to a powder overnight. The powder was dissolved in DMF and purified via preparative LC-MS.

Synthesis of 2,2,2-Trifluoroethyl-2-glycylhydrazine-1-carboxylate.

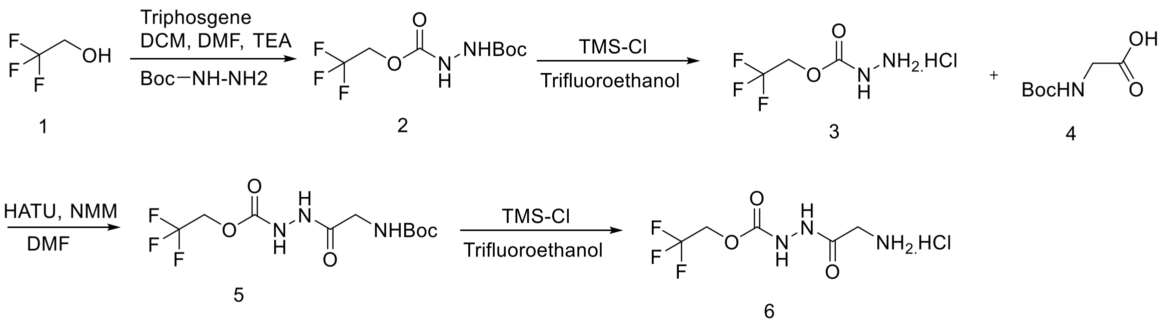

1-(*tert*-Butyl) 2-(2,2,2-trifluoroethyl) hydrazine-1,2-dicarboxylate (2): To a stirred solution of 2,2,2-trifluoroethanol 1 (7.27 mL,101.09 mmol), triphosgene (10 g, 33.7 mmol), and dichloromethane (120 mL), Triethyl amine (14.07 mL, 101.09 mmol) was added dropwise at 0 °C for 10 min and stirred for 4 h at RT. Then, the mixture was filtered and washed with 2 x 20 mL of dichloromethane. The filtrate was then added dropwise into another round bottom flask which was equipped with a magnetic stir bar and charged with *tert*-butyl hydrazine carboxylate (13.36 g, 101.09 mmol), triethylamine (35.20 mL, 252.67mmol), and dichloromethane (100 mL) at 0 °C for 1h. Then the reaction mixture was stirred at 0 °C for another 30 min and then 3 h at RT. After completion of reaction, water (100 mL) was added to the reaction mixture, the aqueous and organic phases were separated. The organic layer was washed with 1M HCl (2 x 50 mL), the organic layer was dried with sodium sulfate, filtered, and concentrated under reduced pressure to get crude residue which was purified by column chromatography (10-20 % EtOAc in hexanes as the eluent) to afford 2 as a white solid (8.35 g, 96 %). ^{1}H NMR (400 MHz, DMSO) δ 9.45 (s, 1H), 8.93 (s, 1H), 4.70 (q, J = 9.1 Hz, 2H), 1.40 (s, 9H). ^{13}C NMR (101 MHz, DMSO) δ 155.45, 155.05, 127.75, 124.99, 122.23, 119.47, 79.50, 60.67, 60.32, 59.97, 59.62, 28.05. ^{19}F NMR (377 MHz, DMSO) δ -72.85.

2,2,2-Trifluoroethyl hydrazinecarboxylate hydrochloride (3): To a solution of compound 2 (8.3 g, 32.14 mmol) in trifluoroethanol (66.4 mL) was added trimethylsilyl chloride (12.45 mL) slowly at

0 °C, and the resulting solution was stirred for 2 h at RT under an inert condition. After completion of the reaction, as indicated by TLC, solvent was removed under reduced pressure to yield compound 3 as a white solid (6.06 g, 97 %). ^{1}H NMR (400 MHz, DMSO) δ 10.66 (d, J = 215.8 Hz, 3H), 4.82 (q, J = 9.0 Hz, 2H). ^{13}C NMR (101 MHz, DMSO) δ 154.18, 127.52, 124.76, 122.00, 119.24, 61.48, 61.13, 60.78, 60.43. 19F NMR (377 MHz, DMSO) δ -72.80.

2,2,2-Trifluoroethyl 2-((tert-butoxycarbonyl)glycyl)hydrazine-1-carboxylate (5): To a stirred solution of 3 (6.0 g, 30.8 mmol) and 4 (6.47 g, 36.93 mmol) in anhydrous DMF (64 mL) was added NMM (9.35 g, 92.4 mmol) slowly and cooled to 0 °C. HATU (17.56 g 46.2 mmol) was added to the reaction mass at 0 °C and then the reaction mixture was warmed to RT and stirred for 12 h. The reaction mixture was then diluted with ethyl acetate (100 mL) and washed with saturated $NaHCO_3$ solution (2 x 50 mL), 1 M HCl solution (2 x 50 mL), and saturated brine solution (2 x 50 mL) sequentially. The organic layer was dried over anhydrous Na_2SO_4 and then concentrated *in vacuo*. The residue was then purified by flash chromatography (50-80 % EtOAc in hexanes as the eluent) to afford 5 as white solid (7.76 g, 80 %). ^{1}H NMR (400 MHz, DMSO) δ 9.85 (s, 1H), 9.60 (s, 1H), 7.02 (t, J = 6.2 Hz, 1H), 4.71 (q, J = 9.1 Hz, 2H), 3.57 (d, J = 6.2 Hz, 2H), 1.38 (s, 9H). ^{19}F NMR (377 MHz, DMSO) δ -72.82.

2,2,2-Trifluoroethyl 2-glycylhydrazine-1-carboxylate hydrochloride (6): To a solution of compound 5 (7.5 g, 23.79 mmol) in trifluoroethanol (60 mL) was added trimethylsilyl chloride (11.25 mL) slowly at 0 °C, and the resulting solution was stirred for 2 h at RT under an inert condition. After completion of the reaction, as indicated by TLC, solvent was removed under reduced pressure to yield compound 6 as white solid (5.38 g, 90 %). ^{1}H NMR (400 MHz, DMSO) δ 10.52 (s, 1H), 9.86 (s, 1H), 8.33 (s, 3H), 4.74 (q, J = 9.0 Hz, 2H), 3.62 (s, 2H). ^{13}C NMR (101 MHz, DMSO) δ 166.05, 154.33, 127.73, 124.97, 122.21, 119.45, 60.84, 60.48, 60.14, 59.79, 38.90. ^{19}F NMR (377 MHz, DMSO) δ -72.83. HRMS (ESI) calculated for $C_5H_9F_3N_3O_3$ 216.0591, found 216.0587.

Synthesis of 2-(7-(2-aminoacetamido)-2-oxo-2H-chromen-4-yl) acetic acid.

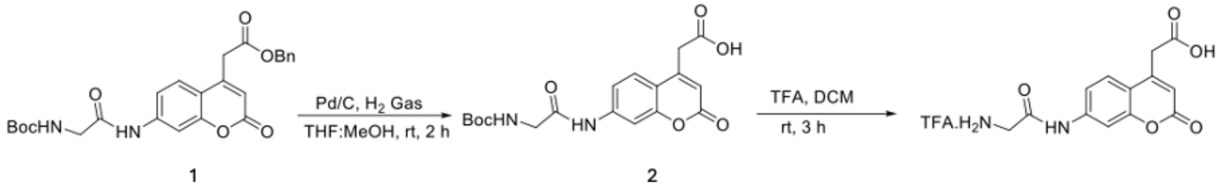

According to ref.1, benzyl 2-(7-(2-((tert-butoxycarbonyl)amino)acetamido)-2-oxo-2H-chromen-4-yl)acetate (1) was synthesized.

2-(7-(2-((tert-butoxycarbonyl)amino)acetamido)-2-oxo-2H-chromen-4-yl)acetic acid (2): To a stirred solution of 1 (2.0 g, 3.43 mmol) in THF: Methanol (1:1, 20 mL) was added 10% Pd/C (160 mg). The reaction flask was sealed and the air inside was removed under the Schlenk line. A hydrogen balloon was then attached to the reaction flask and the reaction mixture was stirred at room temperature for 2 h. After the completion of deprotection, the reaction mixture was filtered with a pad of Celite. The filtrate was concentrated in vacuo and the residue was used for the next step without further purification.

2-(7-(2-aminoacetamido)-2-oxo-2H-chromen-4-yl)acetic acid: To a stirred solution of 2 (1.50 g, 3.98 mmol) in anhydrous DCM (10 mL) at 0 °C was added TFA (3 mL). The reaction mixture was stirred at room temperature for 3 h. After completion of the reaction, remove the solvent in vacuo and wash with hexane to afford SR-D-35 as a white solid (1.0 g, 90%). 1H NMR (400 MHz, DMSO) δ 7.80 (t, J = 5.3 Hz, 1H), 7.72 (d, J = 8.7 Hz, 1H), 7.44 (d, J = 8.8 Hz, 1H), 6.41 (s, 1H), 3.89 (s, 2H), 3.86 (s, 2H).

Acknowledgments

This work was supported by the Welch Foundation (grant A-1715 to W.R.L.) and National Institutes of Health (grants R35GM145351 to W.R.L., R01GM129076 to A.J.W., R01CA251677 to Y.Z., and P30CA014599 to U. Chicago). The authors thank Mr. Yohannes Rezenom in the Mass Spectrometry Facility of Texas A&M University for helping with running the LC-MS characterizations of all proteins.

References

1. Heiner, S.; Heiner, D.; Kuhn, A.; Kunz, H., Hydrophilic photolabelling of glycopeptides from murine liver-intestine (LI) cadherin recognition domain. *Bioorganic and Medicinal Chemistry* **2006,** 14 (18), 6149-6164.

Supplementary Figures

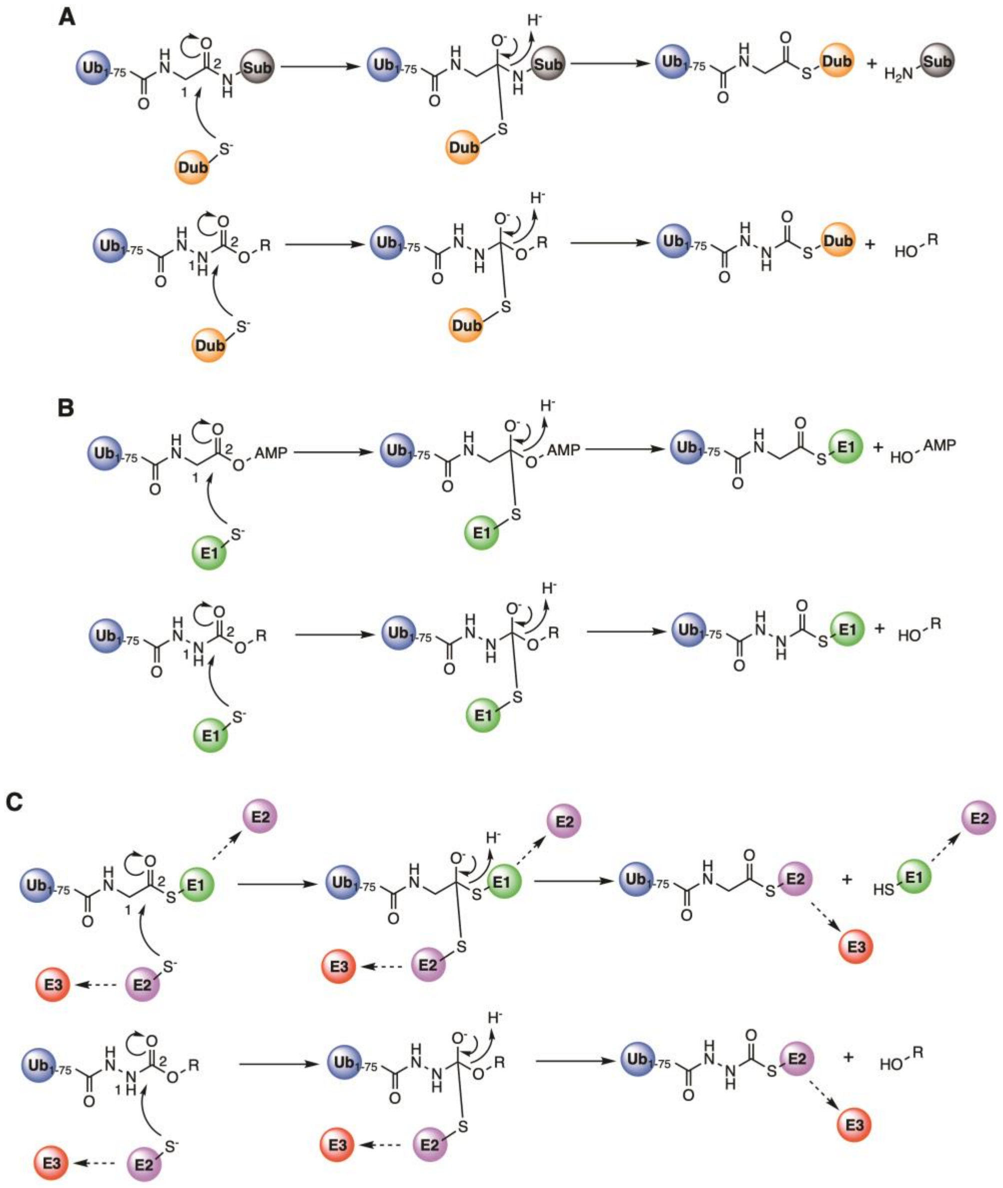

Figure S1. Ub azapeptide ester probes react with (**A**) DUB, (**B**) E1, (**C**) E2, and (**C**) E3 catalytic cysteines via a transition state mimicking that in reactions catalyzed by the four enzyme groups. All reactions pass through a tetrahedral transition state that is potentially stabilized by an anion hole in the enzyme active site.

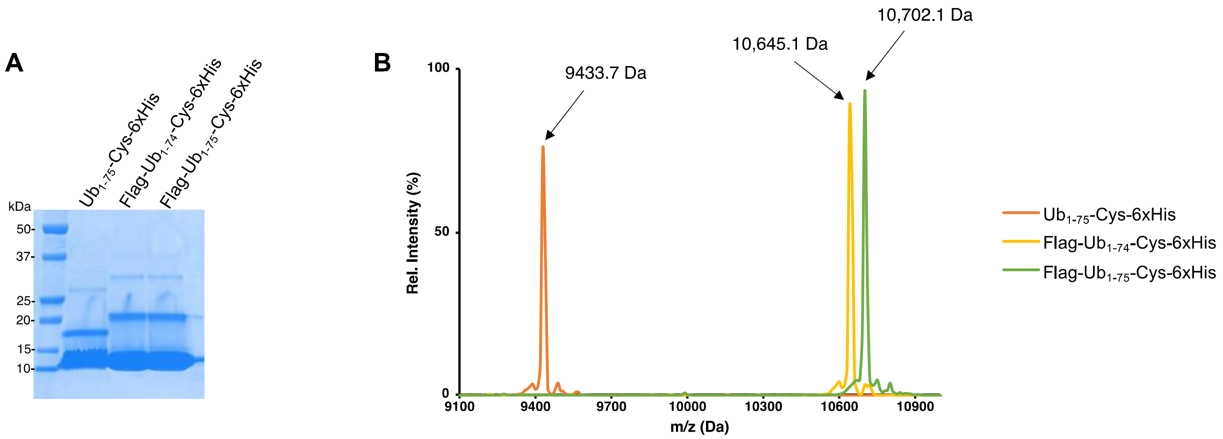

Figure S2. (**A**) SDS-PAGE and Coomassie blue staining of $Ub_{1\text{-}75}$-Cys-6×His, Flag-$Ub_{1\text{-}74}$-Cys-6×His, and Flag-$Ub_{1\text{-}75}$-Cys-6×His; (**B**) Deconvoluted ESI-MS spectra of $Ub_{1\text{-}75}$-Cys-6×His, Flag-$Ub_{1\text{-}74}$-Cys-6×His, and Flag-$Ub_{1\text{-}75}$-Cys-6×His. Arrow-indicated values represent detected molecular weights of corresponding proteins.

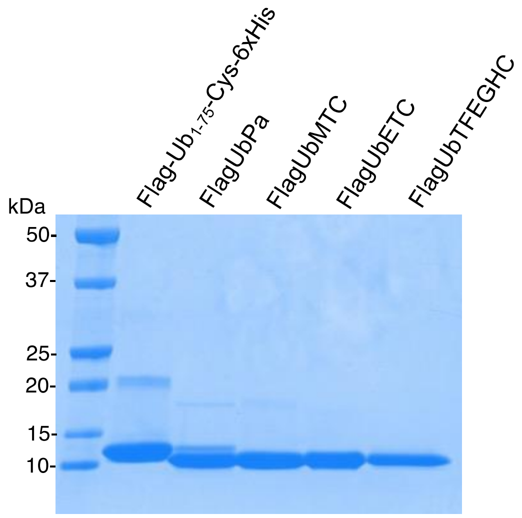

Figure S3. SDS-PAGE and Coomassie blue staining of Flag-Ub$_{1-75}$-Cys-6×His, FlagUbPa and three ubiquitin azapeptide ester probes.

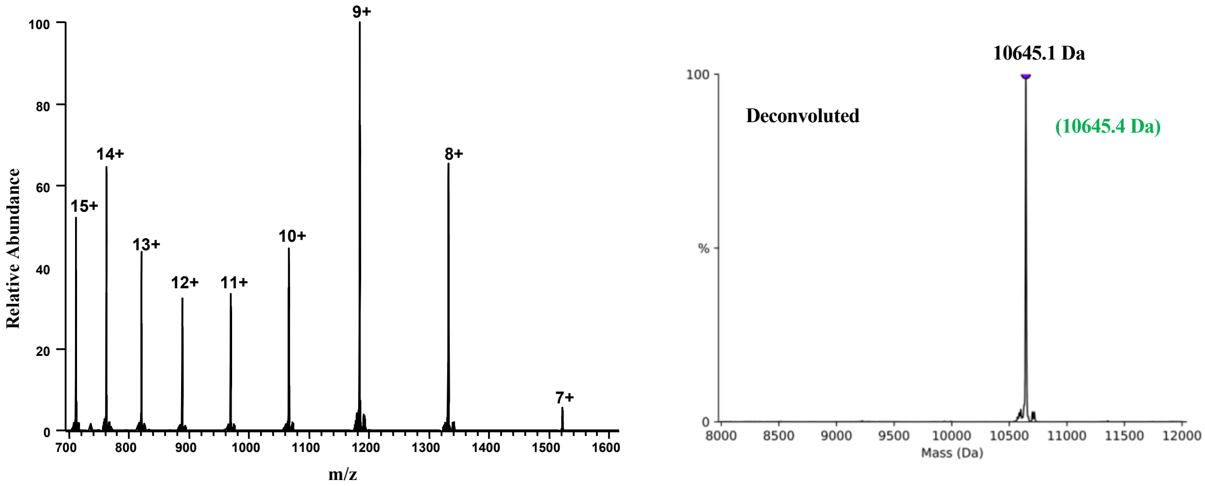

Figure S4. ESI-MS spectra and deconvoluted mass of Flag-Ub$_{1\text{-}74}$-Cys-6×His. The value in green indicates the actual theoretical mass of the protein.

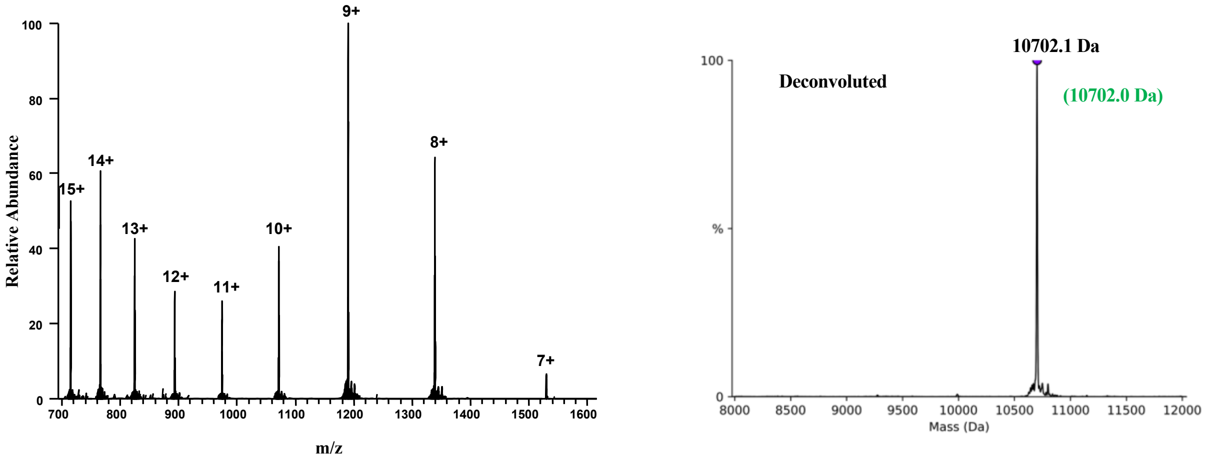

Figure S5. ESI-MS spectra and deconvoluted mass of Flag-Ub$_{1\text{-}75}$-Cys-6×His. The value in green indicates the actual theoretical mass of the protein.

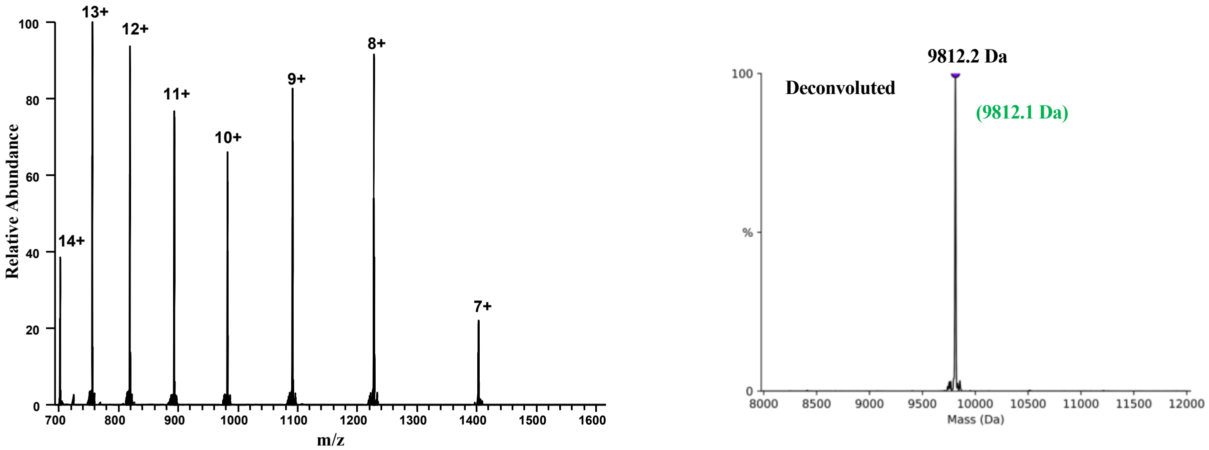

Figure S6. ESI-MS spectra and deconvoluted mass of FlagUbPa. The value in green indicates the actual theoretical mass of the probe.

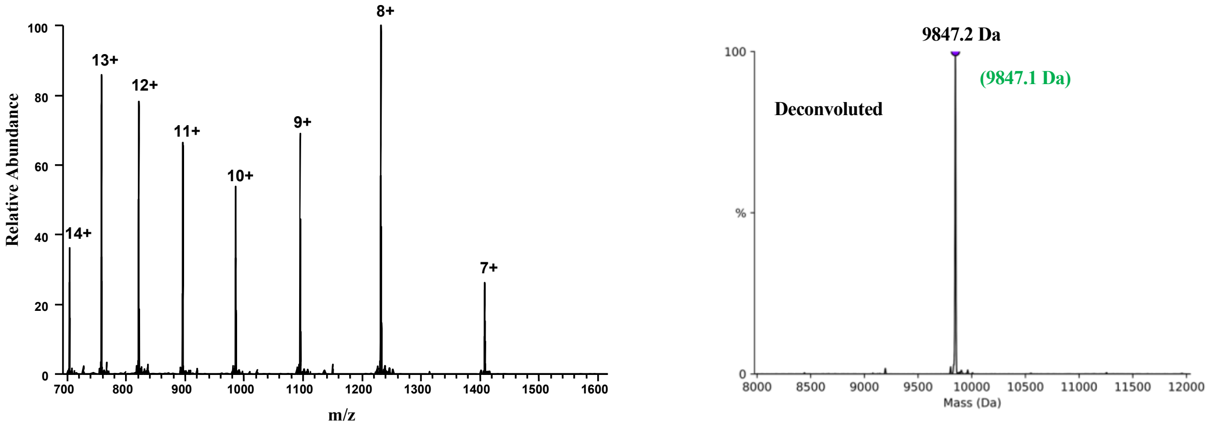

Figure S7. ESI-MS spectra and deconvoluted mass of FlagUbMTC. The value in green indicates the actual theoretical mass of the probe.

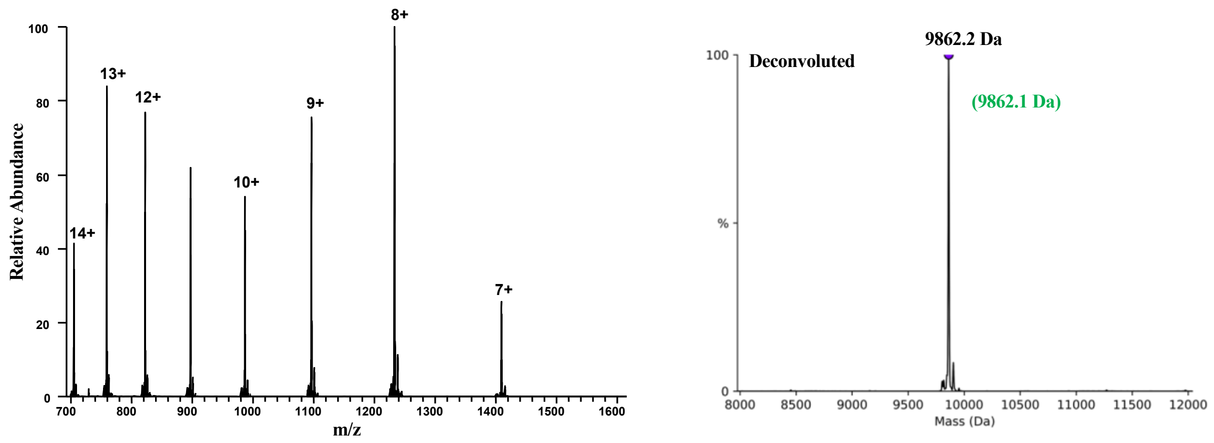

Figure S8. ESI-MS spectra and deconvoluted mass of FlagUbETC. The value in green indicates the actual theoretical mass of the probe.

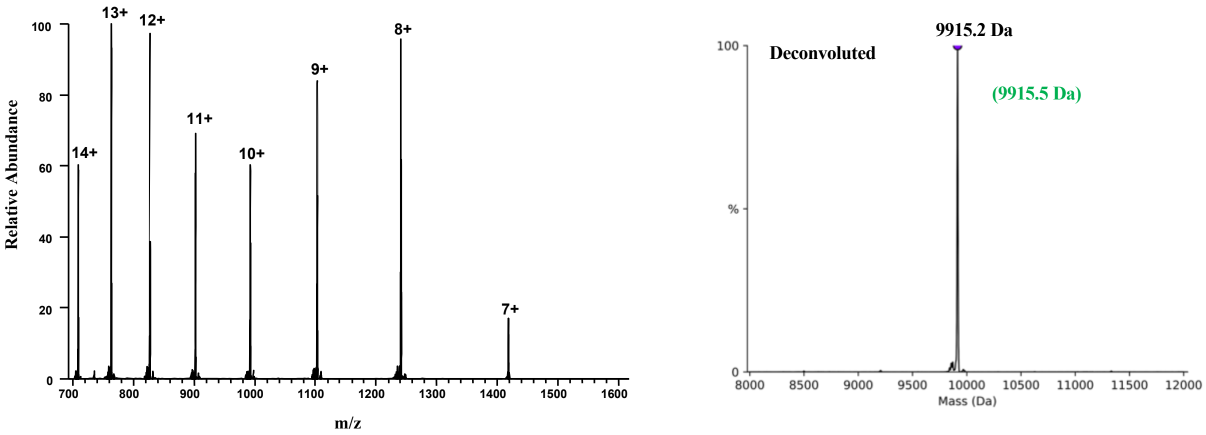

Figure S9. ESI-MS spectra and deconvoluted mass of FlagUbTFEGHC. The value in green indicates the actual theoretical mass of the probe.

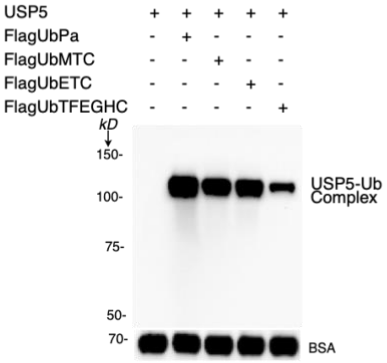

Figure S10. Use of BSA loading control in the reaction between the four Flag fused Ub probes and USP5. The probes and the enzymes were reconstituted in a reaction buffer containing BSA. The BSA was detected using anti-BSA antibody. Since, BSA is not related to the ubiquitination/deubiquitination cascade and also does not have any cross-reactivity, it was used as a loading control for the reaction.

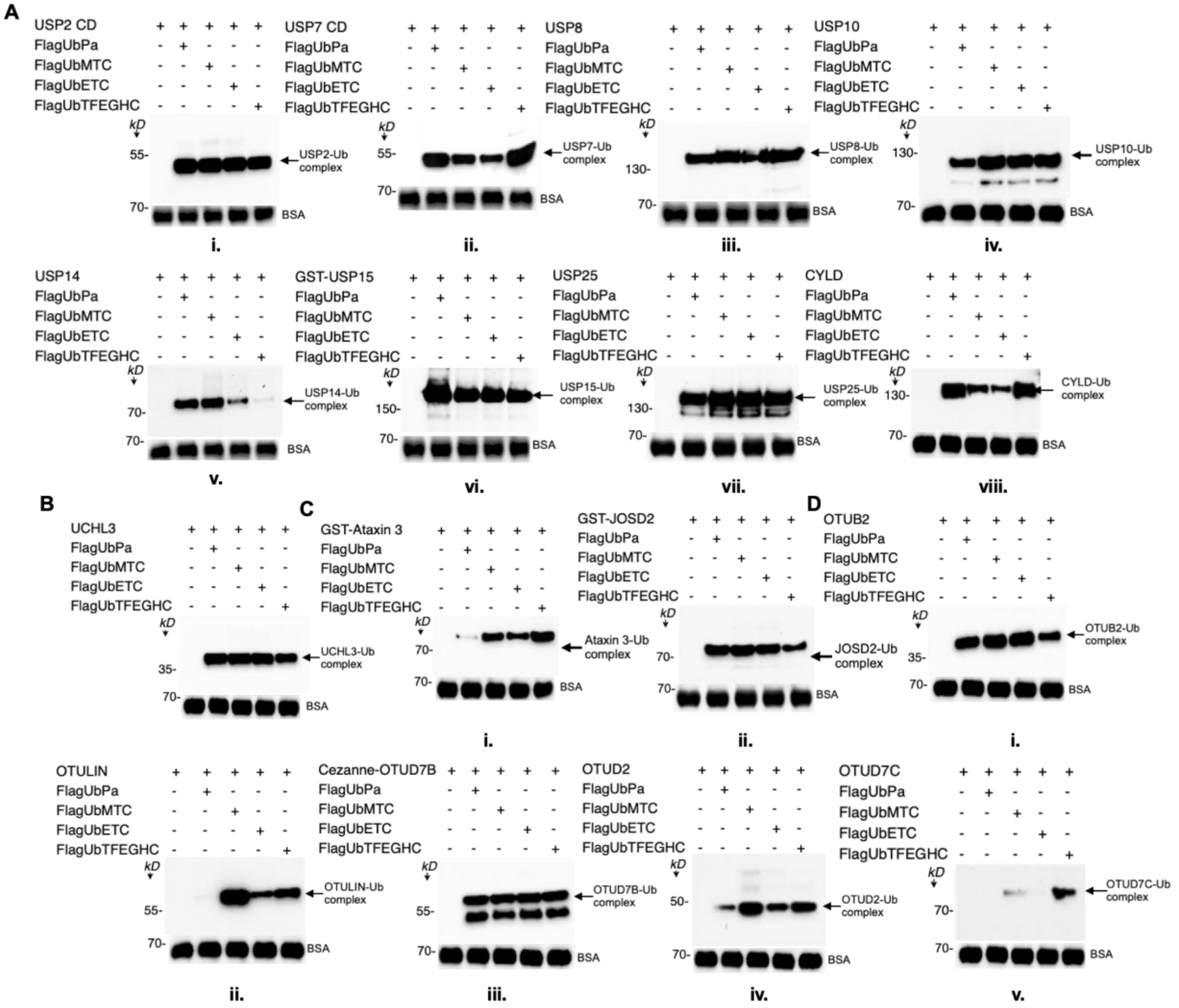

Figure S11. Use of BSA loading control in the reaction between the four Ub probes and DUBs from different superfamily (**A**) USP superfamily (**B**) UCH superfamily (**C**) Josephin superfamily (**D**) OTU superfamily. The probes and the enzymes were reconstituted in a reaction buffer containing BSA. The BSA was detected using anti-BSA antibody. Since, BSA is not related to the ubiquitination/deubiquitination cascade and also does not have any cross-reactivity, it was used as a loading control for the reaction.

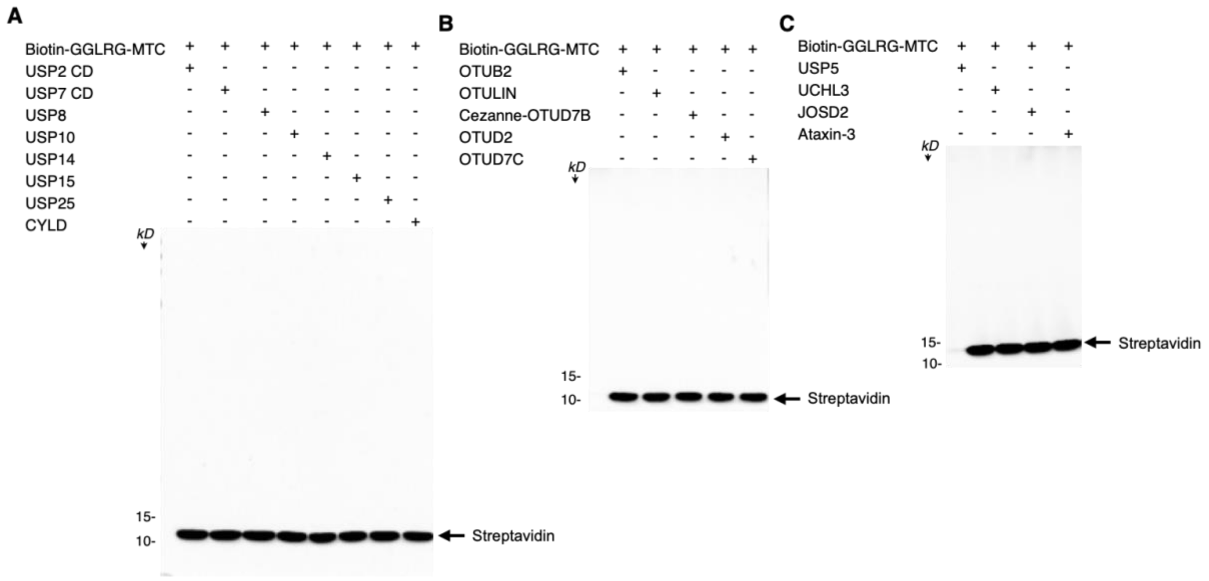

Figure S12. Reaction of Biotin-GGLRG-MTC peptide with the DUBs (**A**) USP superfamily (**B**) OTU superfamily (**C**) UCH and Josephin superfamily. The peptide was incubated with each DUBs from the mentioned superfamilies for 40 minutes at 37 °C. Following the reaction, the reaction was stopped, and the reaction mixture was further incubated with streptavidin. The samples were subsequently analyzed by SDS-PAGE and probed using an anti-streptavidin antibody via western blotting.

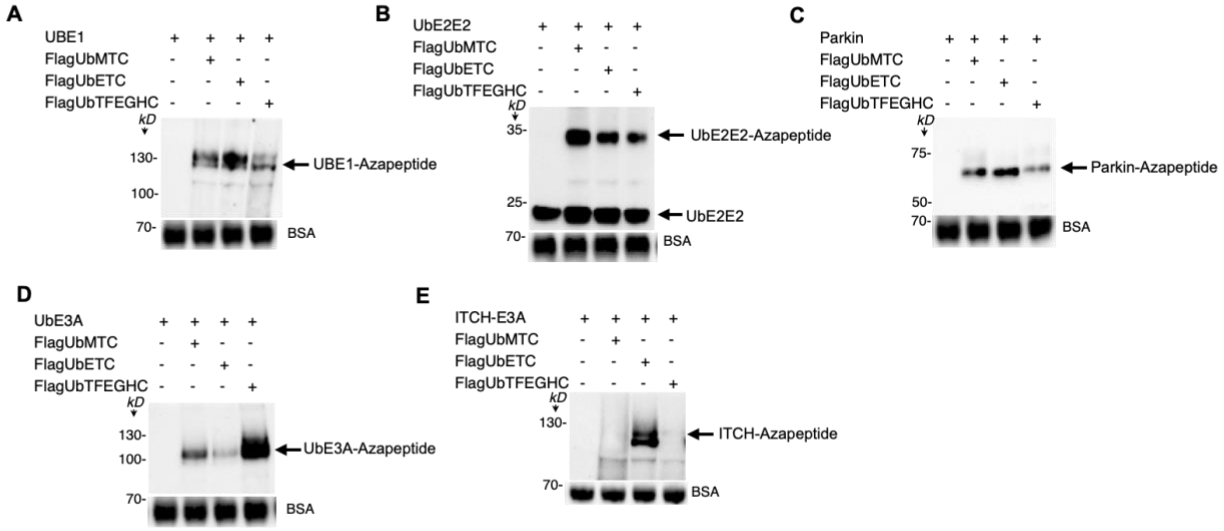

Figure S13. Covalent complex formation between three Ub azapeptide probes and E1, E2 and cysteine-containing E3 enzymes. (**A**) UBE1 (**B**) UbE2E2 (**C**) Parkin (**D**) UbE3A **(E)** ITCH. The probes and the enzymes were reconstituted in a reaction buffer containing BSA. The BSA was detected using anti-BSA antibody. Since, BSA is not related to the ubiquitination/deubiquitination cascade and does not have any cross-reactivity, it was used as a loading control for the reaction.

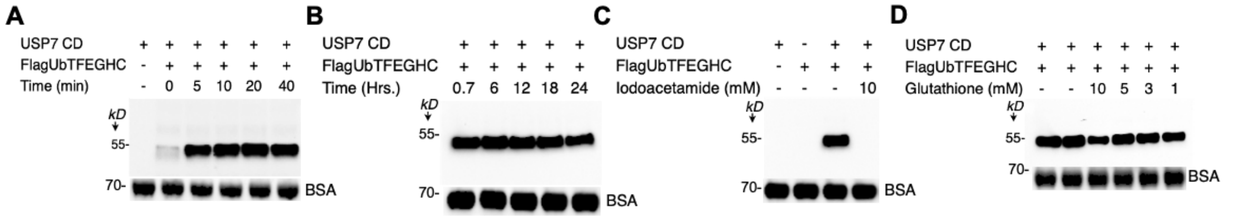

Figure S14. Use of BSA loading control in the characterization of USP7 CD-FlagUbTFEGHC thiocarbamate complex. (**A**) Time dependent assay (**B**) Stability of the thiocarbamate complex over time (**C**) Cysteine labelling experiment, and (**D**) Thiol exchange assay. FlagUbTFEGHC and USP7 CD were reconstituted in a DUB reaction buffer containing BSA. The BSA was detected using anti-BSA antibody. Since, BSA is not related to the ubiquitination/deubiquitination cascade and does not have any cross-reactivity, it was used as a loading control for the reaction.

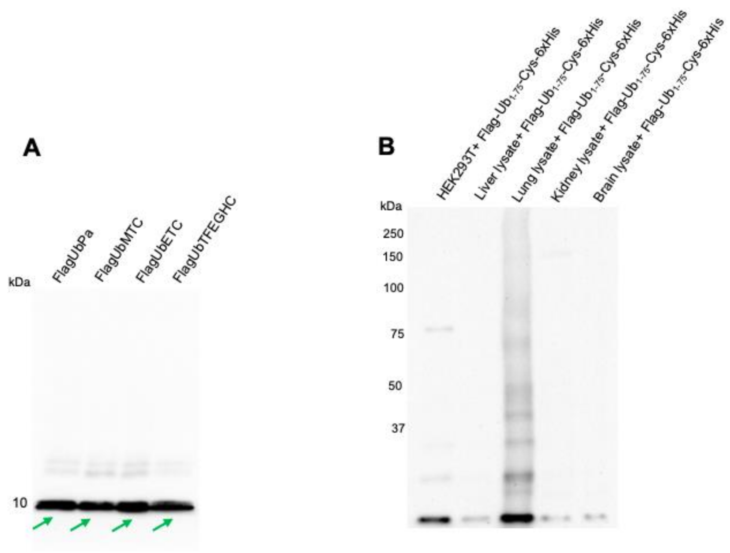

Figure S15. Anti-FLAG detection of (**A**) four ubiquitin probes (**B**) negative control experiments in which HEK293T cell and four mouse tissue lysates were incubated with recombinant Flag-Ub_{1-75}-Cys-6×His.

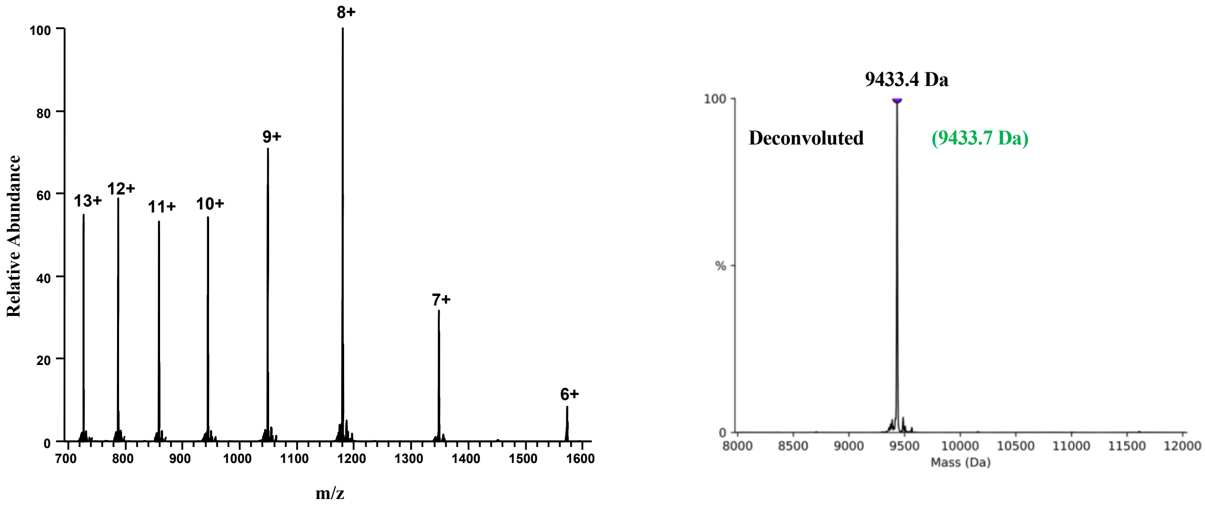

Figure S16. ESI-MS spectra and deconvoluted mass of Ub$_{1\text{-}75}$-Cys-6×His. The value in green indicates the actual theoretical mass of the protein.

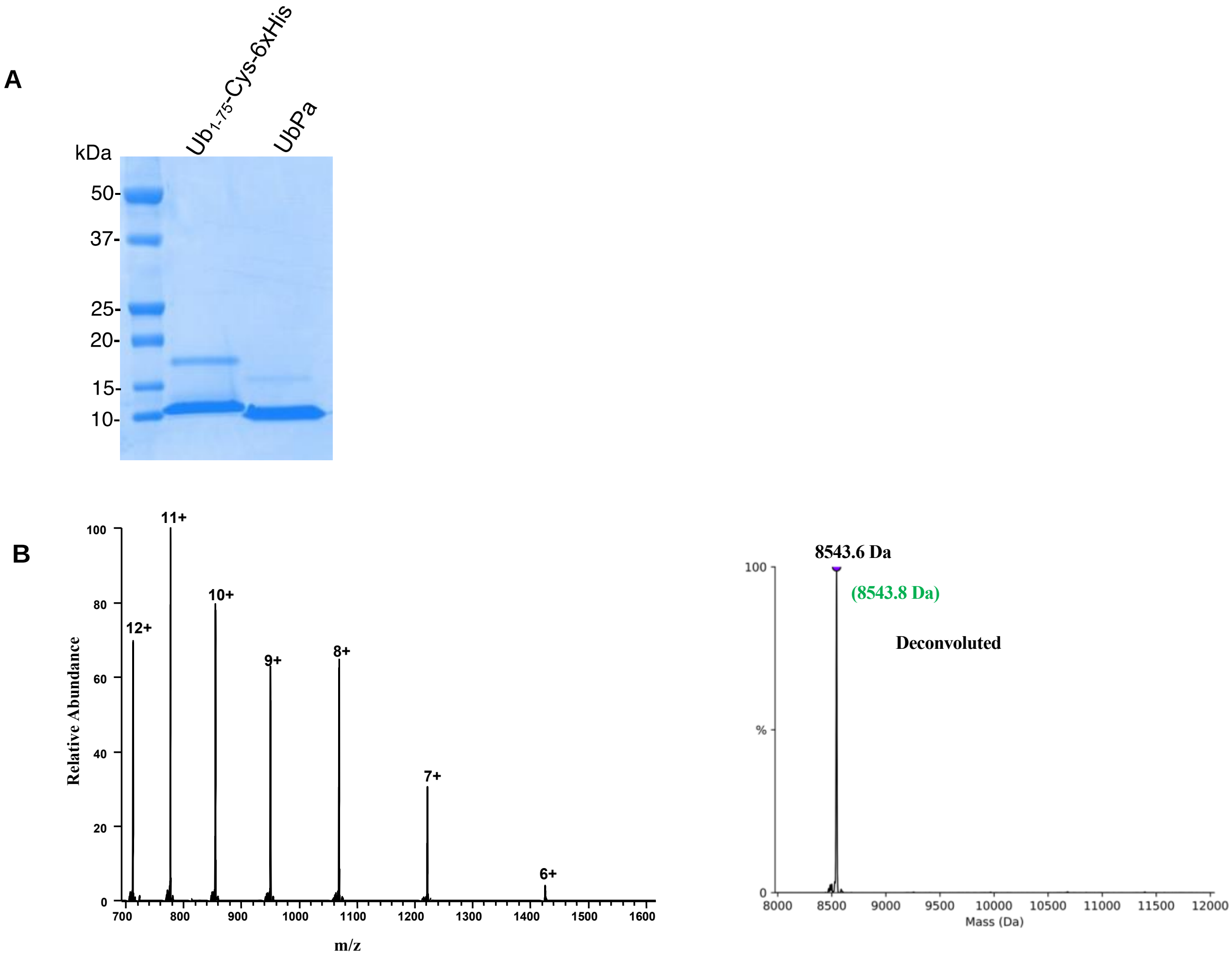

Figure S17. (**A**) SDS-PAGE and Coomassie blue staining of Ub_{1-75}-Cys-6×His and UbPa. (**B**) ESI-MS spectra and deconvoluted mass of UbPa. The value in green indicates the actual theoretical mass of the probe.

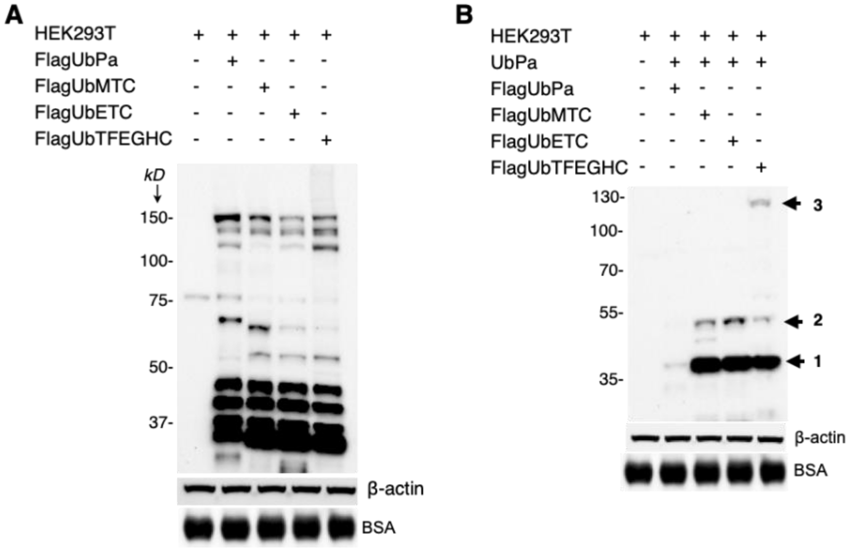

Figure S18. BSA loading control in profiling the cysteine enzymes in the ubiquitination/deubiquitination cascade in HEK293T cell lysates. (**A**) The whole cell lysates treated with FlagUbPa and three Ub azapeptide ester probes (**B**) The whole cell lysates pretreated with UbPa followed by addition of the three Ub azapeptide ester probes. The BSA was detected using anti-BSA antibody. Since, BSA is not related to the ubiquitination/deubiquitination cascade and does not have any cross-reactivity, it was used as a loading control for the reaction.

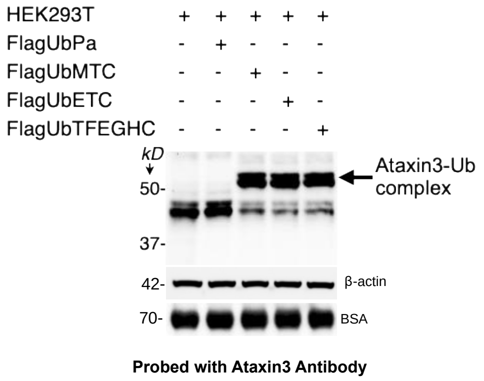

Figure S19. Covalent complex formation between the Ub azapeptide ester probes and ataxin-3 in HEK293T cell lysate. HEK293T Cell lysates were reacted with four probes for 20 hrs at 4°C before their Western blotting analyses by an anti-ataxin-3 antibody. Enzyme-probe complexes that are unique to three ubiquitin azapeptide ester probes are indicated. Unlike the azapeptide ester probes, no covalent complex formation was observed for FlagUbPa.

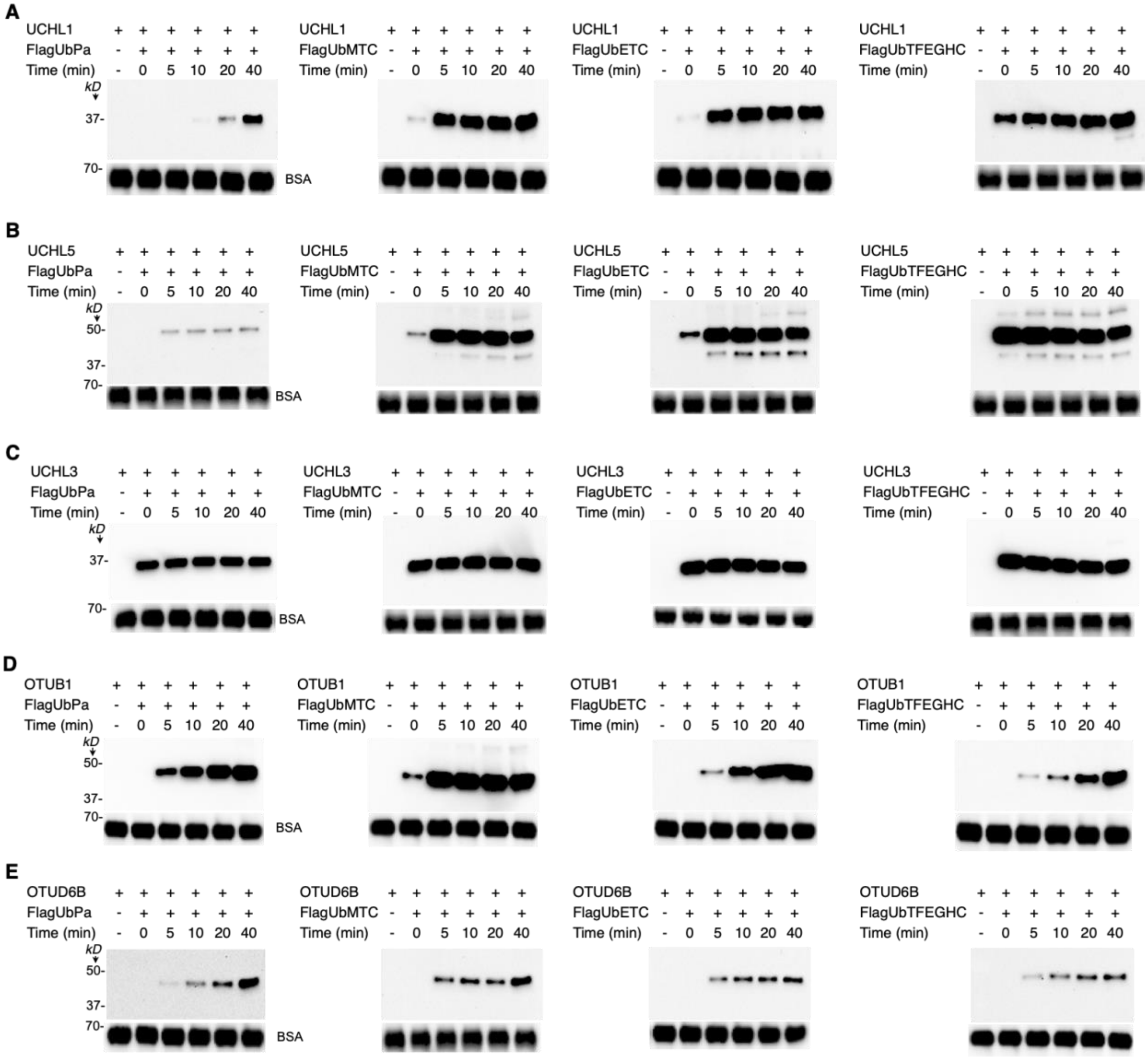

Figure S20. Use of BSA loading control in the kinetic characterization between the four Ub probes and five DUBs such as (**A**) UCHL1 (**B**) UCHL5 (**C**) UCHL3 (**D**) OTUB1, and (**E**) OTUD6B respectively. The probes and the enzymes were reconstituted in a reaction buffer containing BSA. The BSA was detected using anti-BSA antibody. Since, BSA is not related to the ubiquitination/deubiquitination cascade and also does not have any cross-reactivity, it was used as a loading control for the reaction.

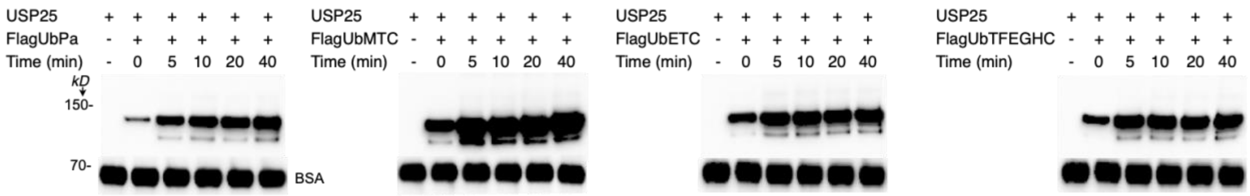

Figure S21. Use of BSA loading control in the time-dependent assay between the four Ub probes and USP25

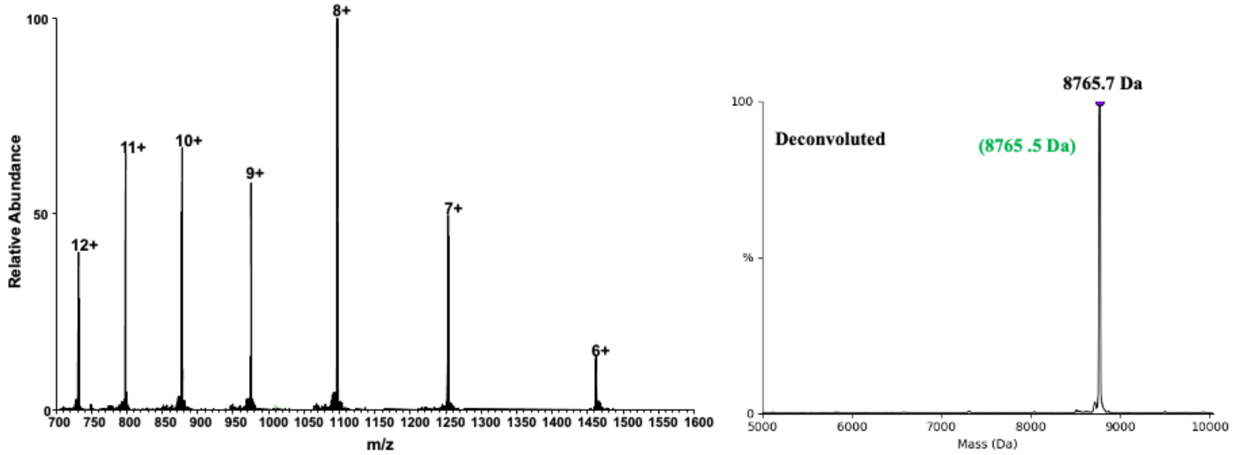

Figure S22. ESI-MS spectra and deconvoluted mass of UbACA. The value in green indicates the actual theoretical mass of the probe.

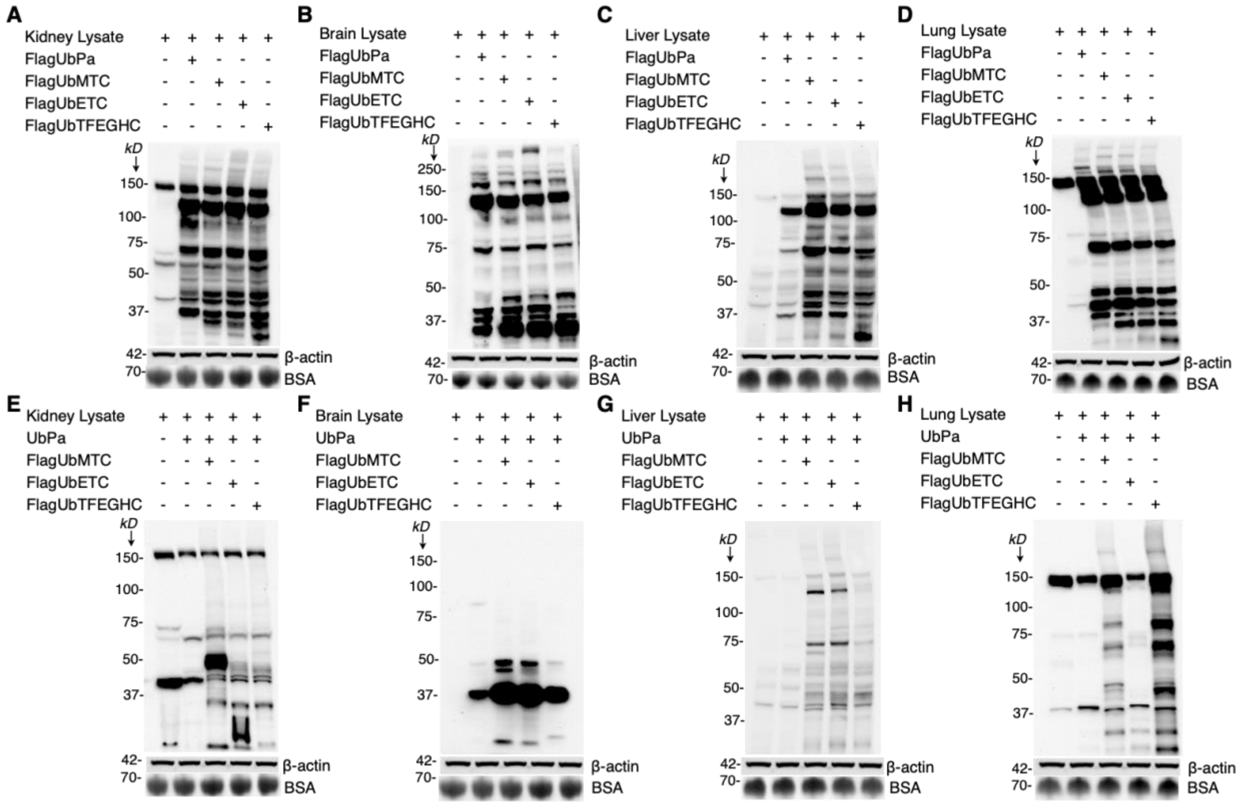

Figure S23. BSA loading control in profiling the cysteine enzymes in the ubiquitination/deubiquitination cascade in different mouse tissues including kidney (**A**), brain (**B**), liver (**C**), and lung (**D**) by four synthetic ubiquitin probes and profiling the same tissues, kidney (**E**), brain (**F**), liver (**G**), and lung (**H**), with three ubiquitin azapeptide ester probes after their reaction with UbPa to remove UbPa-reacting enzymes. The BSA was detected using anti-BSA antibody. Since, BSA is not related to the ubiquitination/deubiquitination cascade and also does not have any cross-reactivity, it was used as a loading control for the reaction.

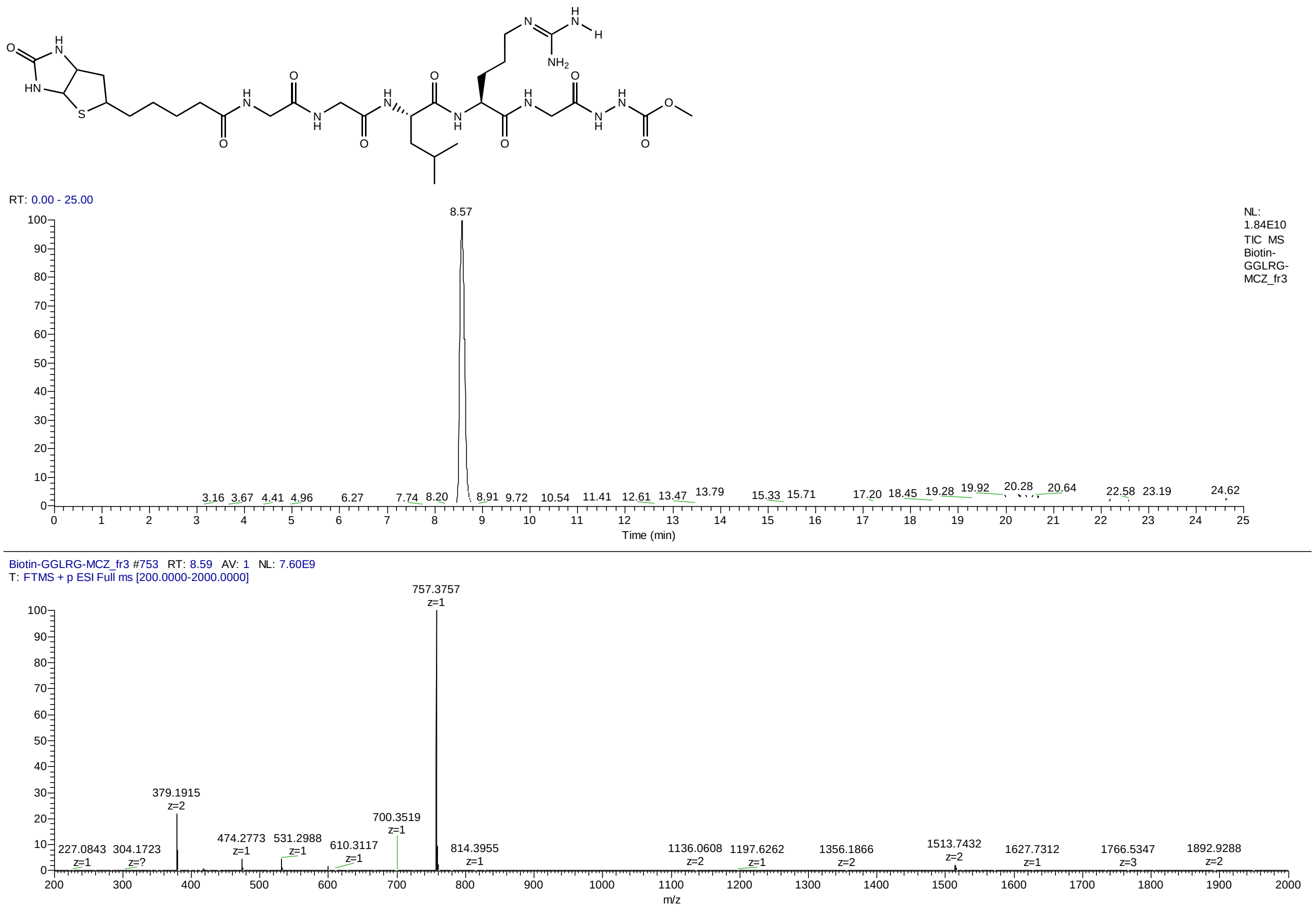

Figure S24. ESI-MS spectra for Biotin-GG-LRG-MTC peptide.

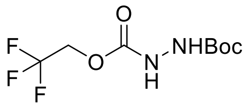

2

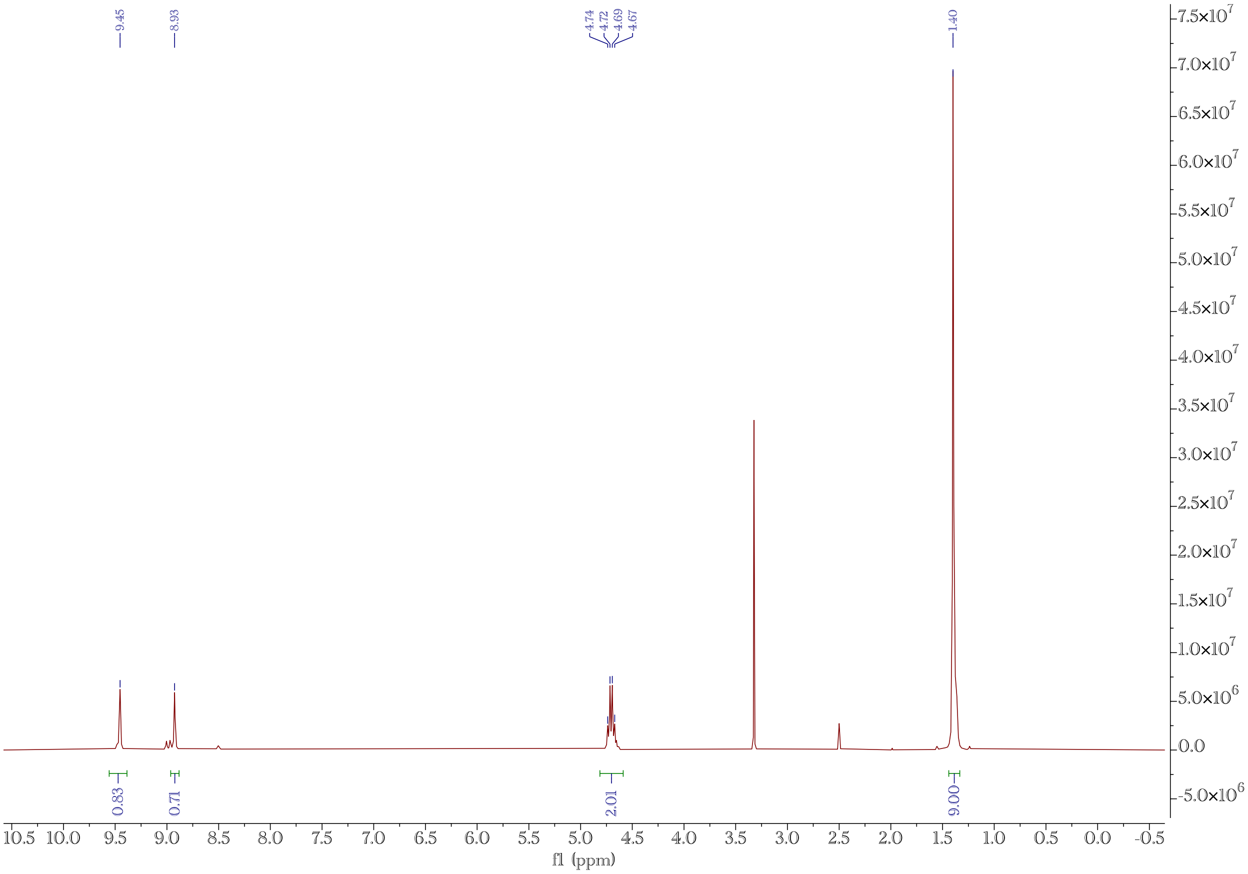

Figure S25. ^{1}H NMR spectrum of Compound 2.

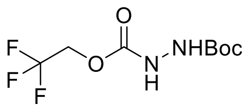

2

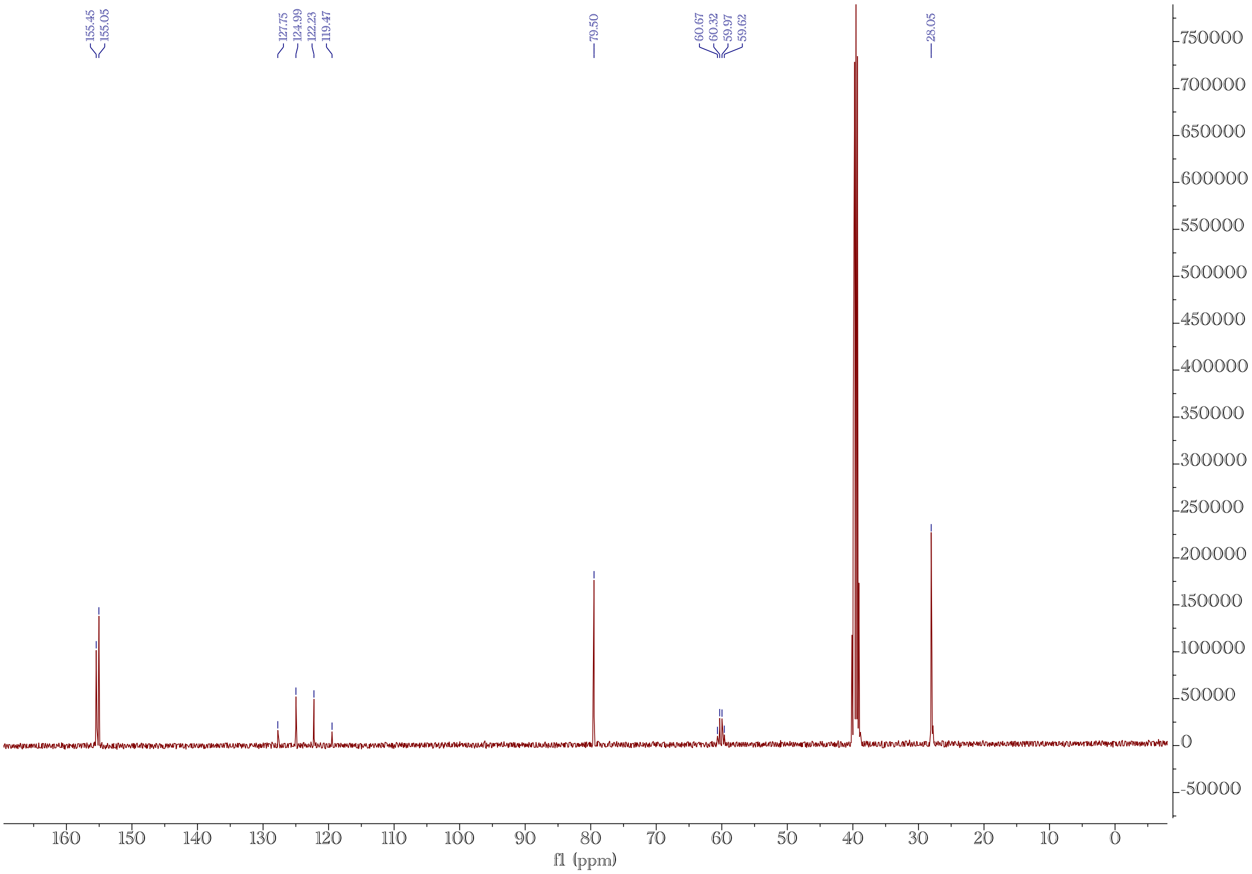

Figure S26. ^{13}C NMR spectrum of Compound 2.

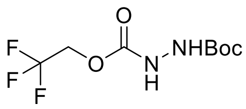

2

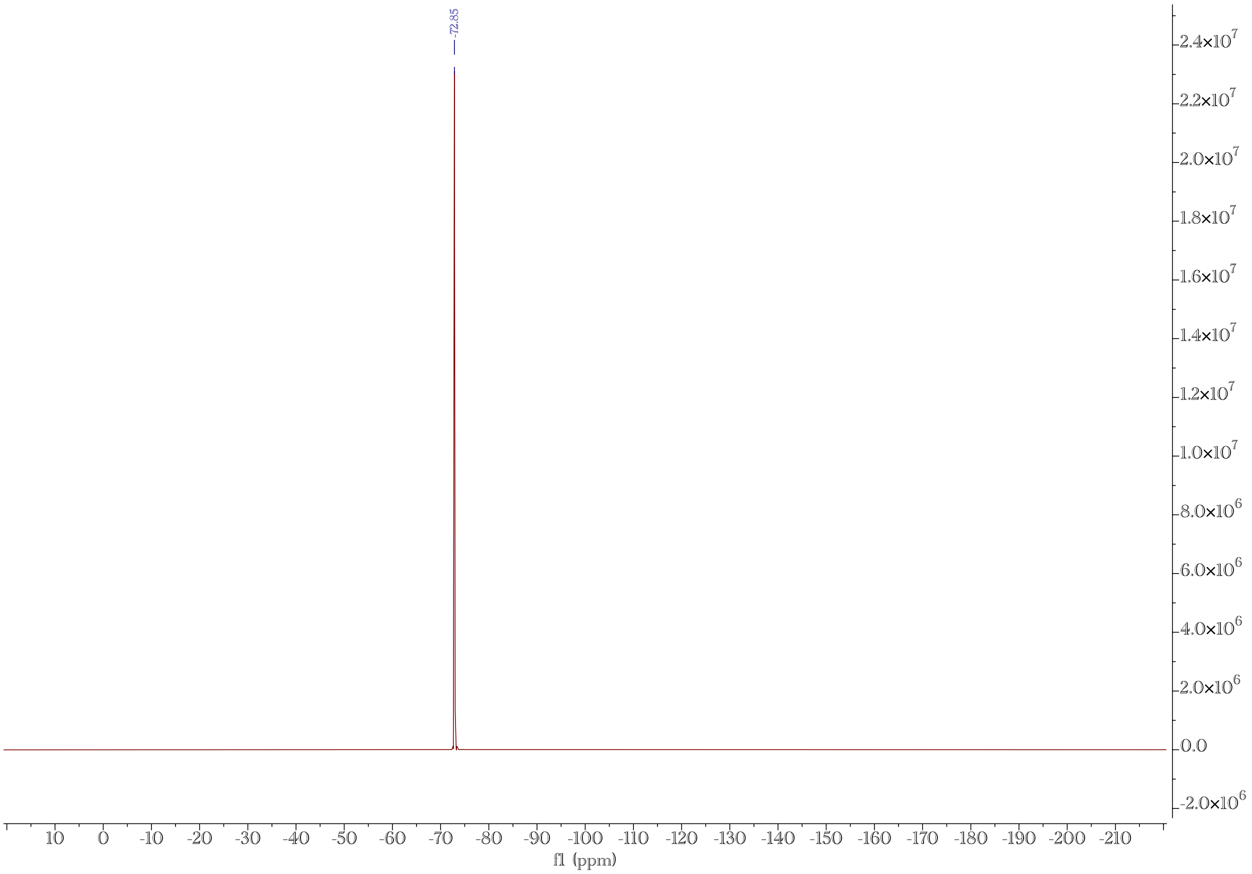

Figure S27. ^{19}F NMR spectrum of Compound 2.

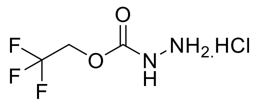

3

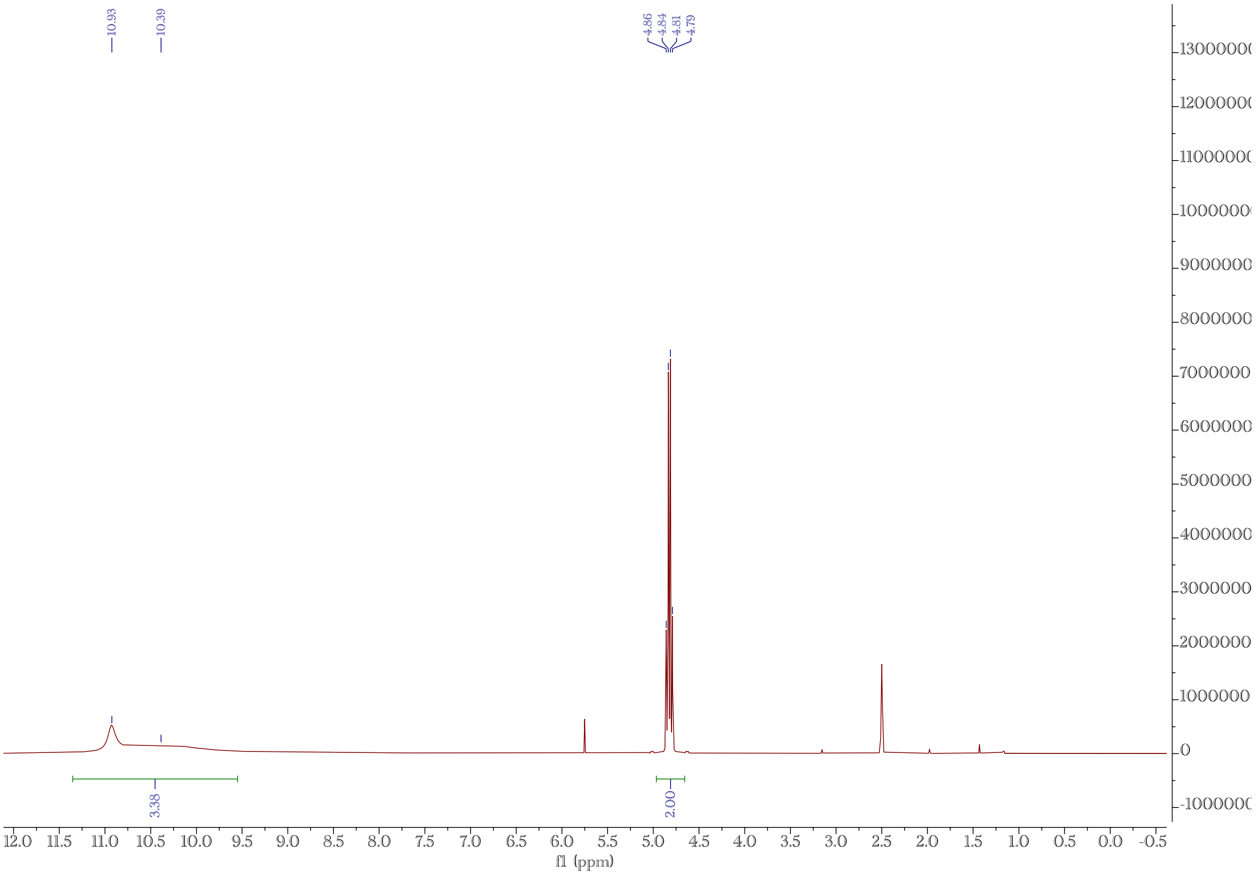

Figure S28. ^{1}H NMR spectrum of Compound 3.

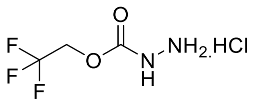

3

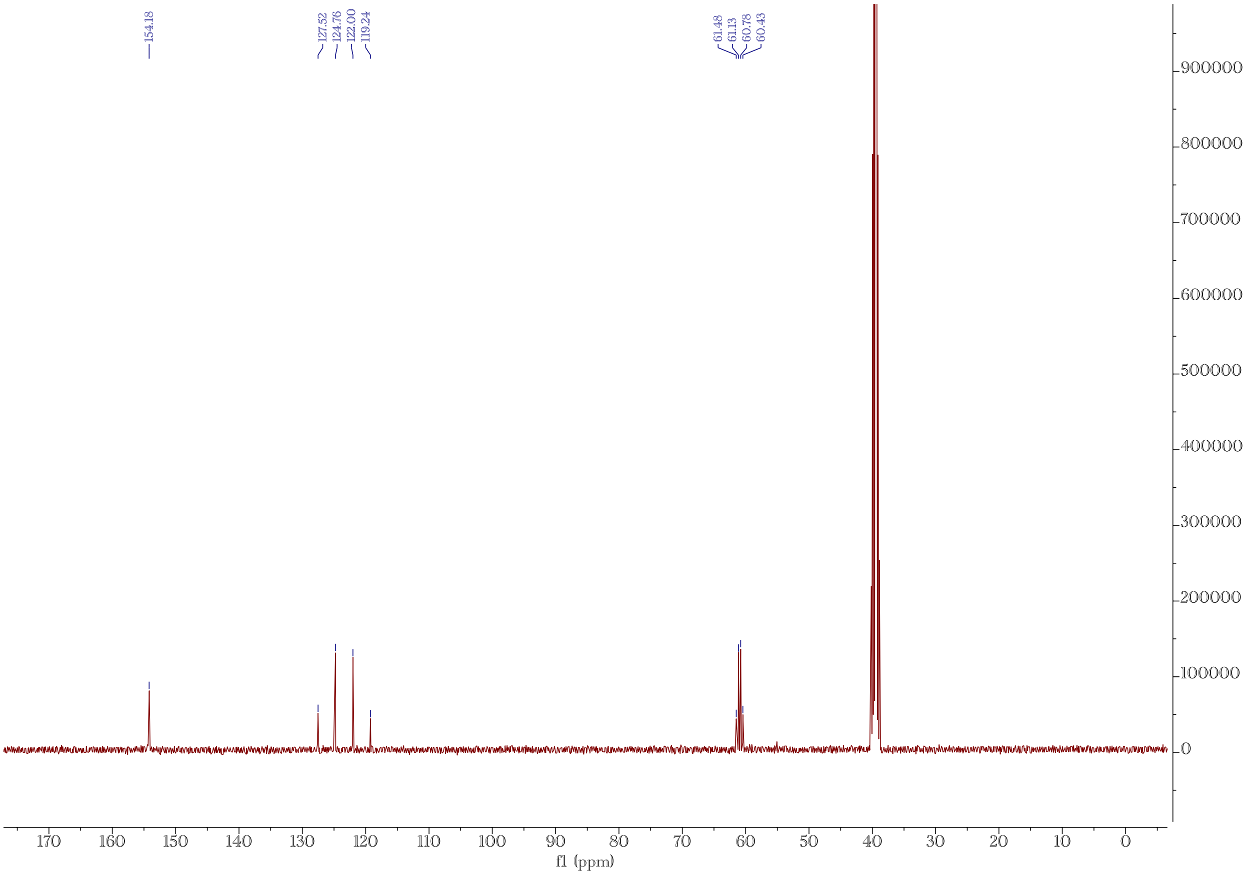

Figure S29. ^{13}C NMR spectrum of Compound 3.

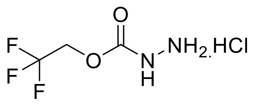

3

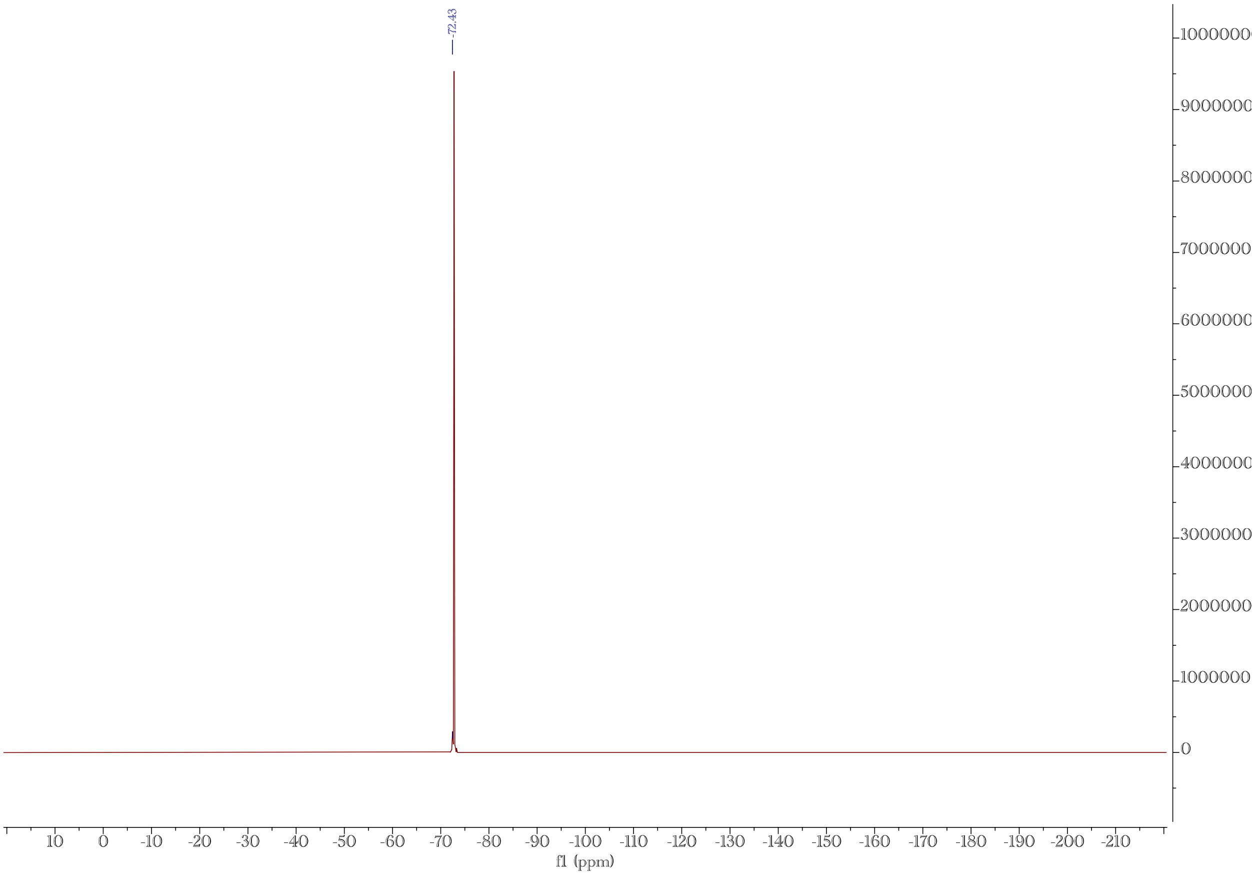

Figure S30. ^{19}F NMR spectrum of Compound 3.

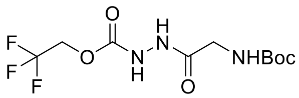

5

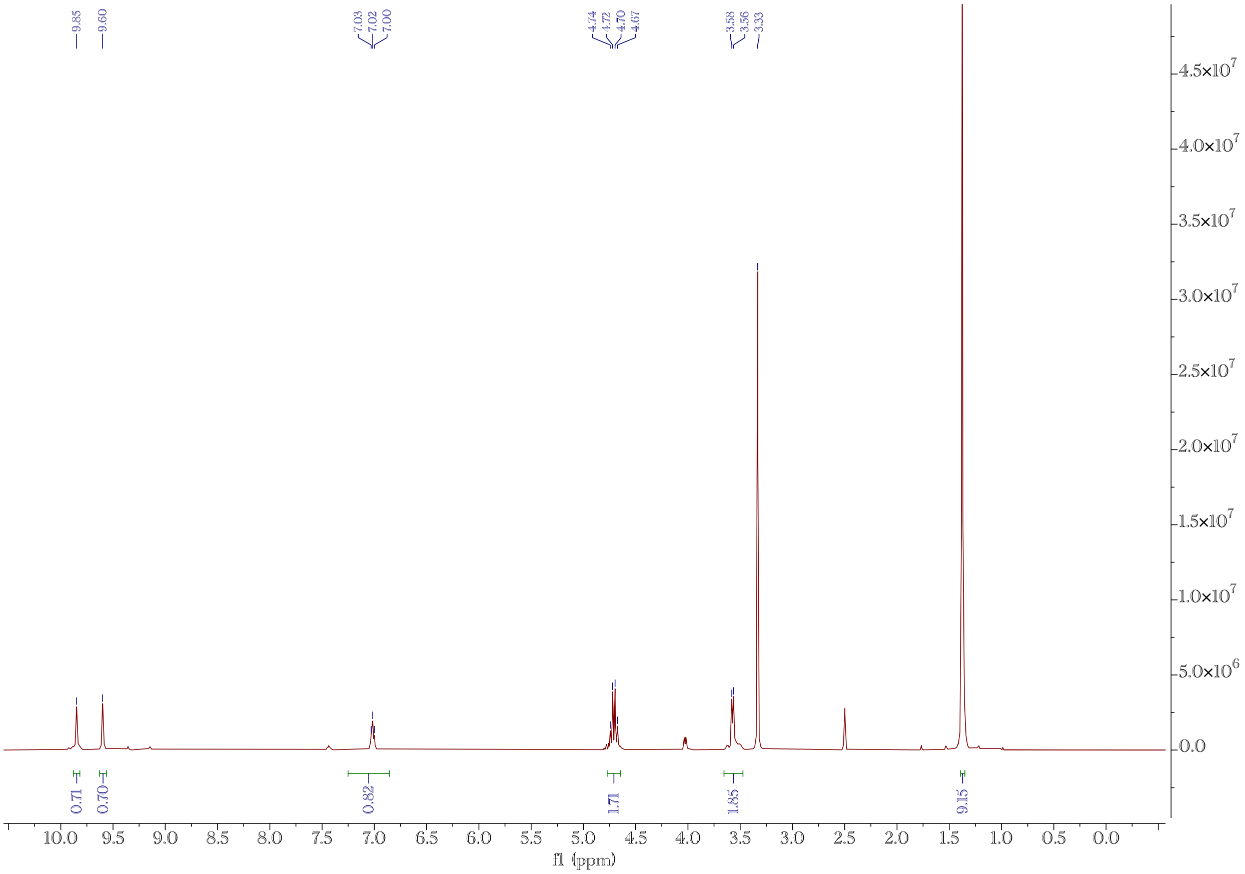

Figure S31. ^{1}H NMR spectrum of Compound 5.

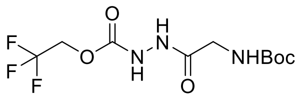

5

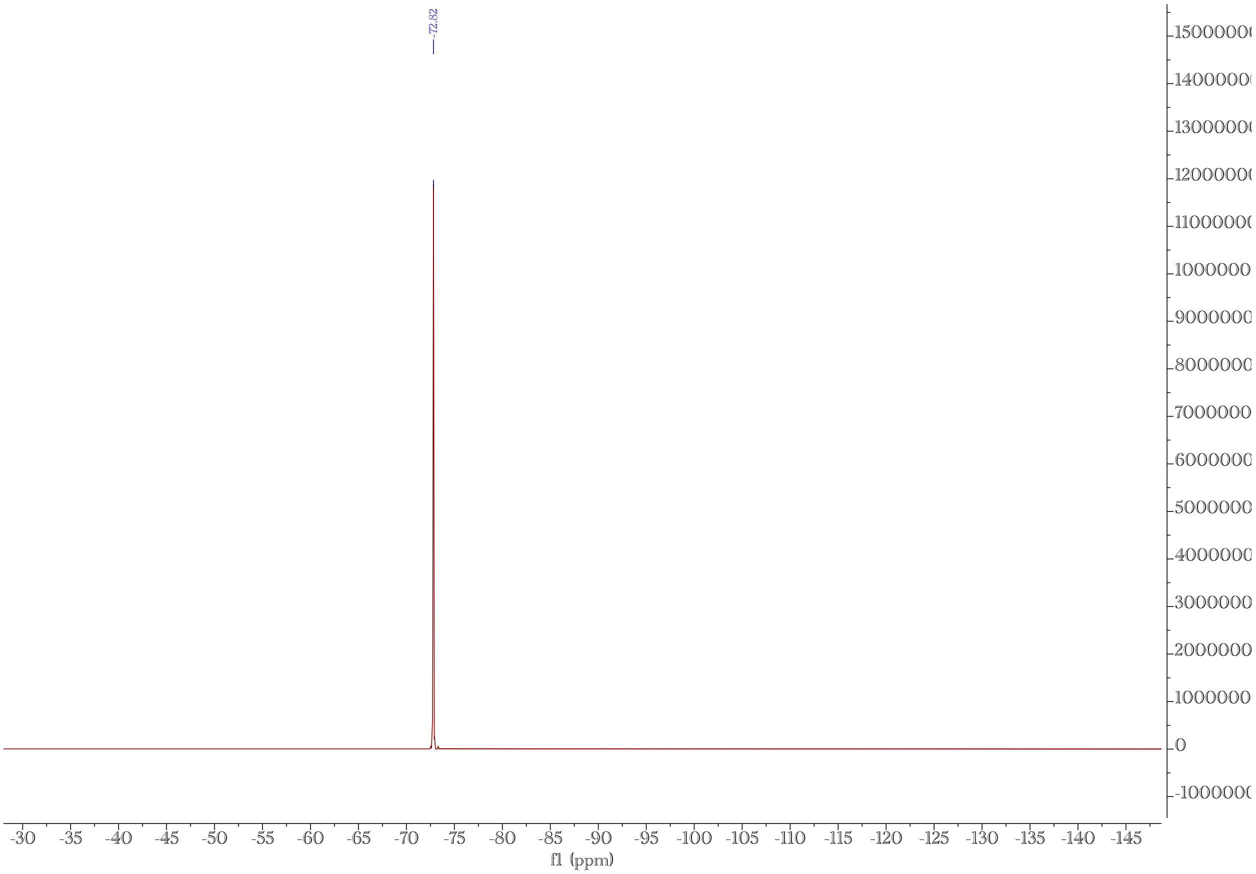

Figure S32. ^{19}F NMR spectrum of Compound 5.

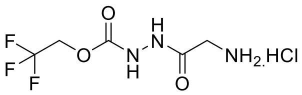

6

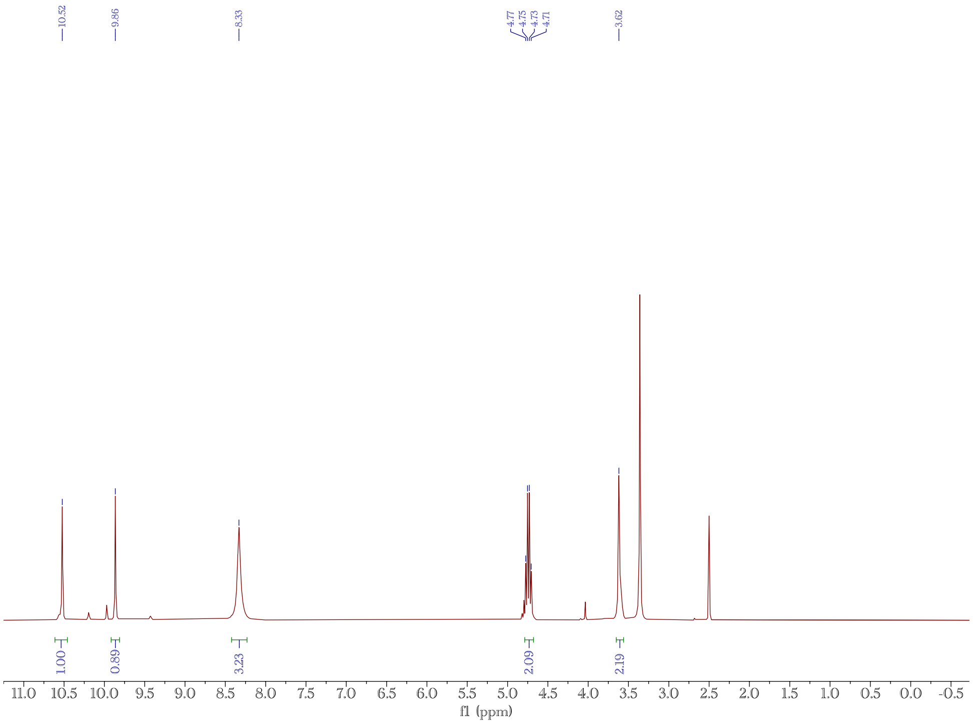

Figure S33. ^{1}H NMR spectrum of Compound 6.

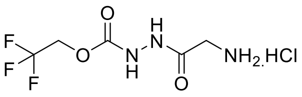

6

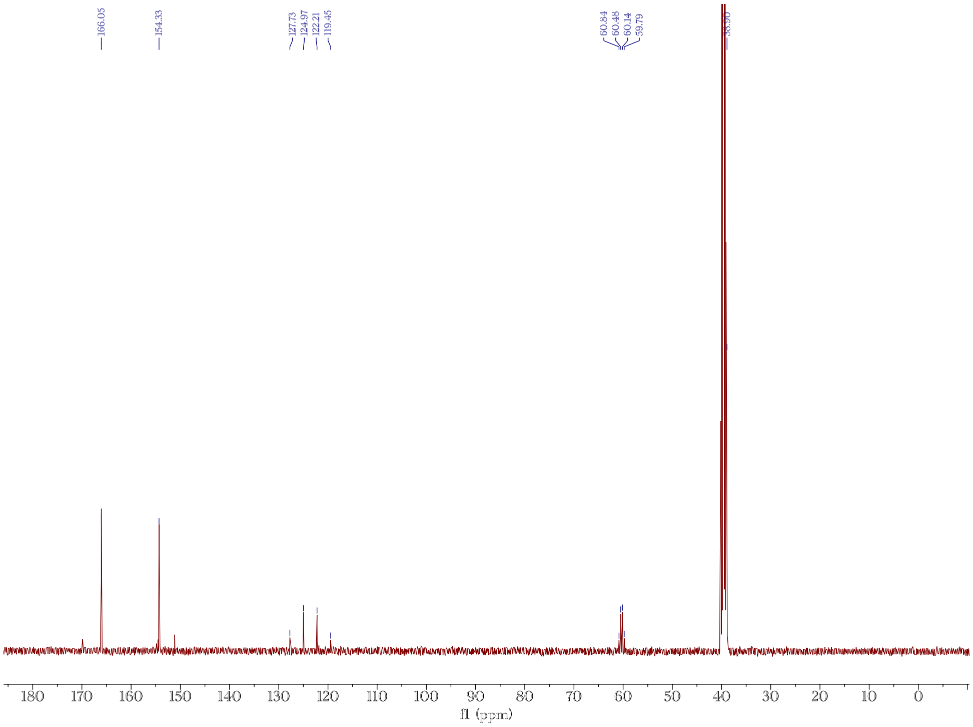

Figure S34. ^{13}C NMR spectrum of Compound 6.

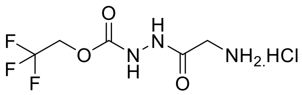

6

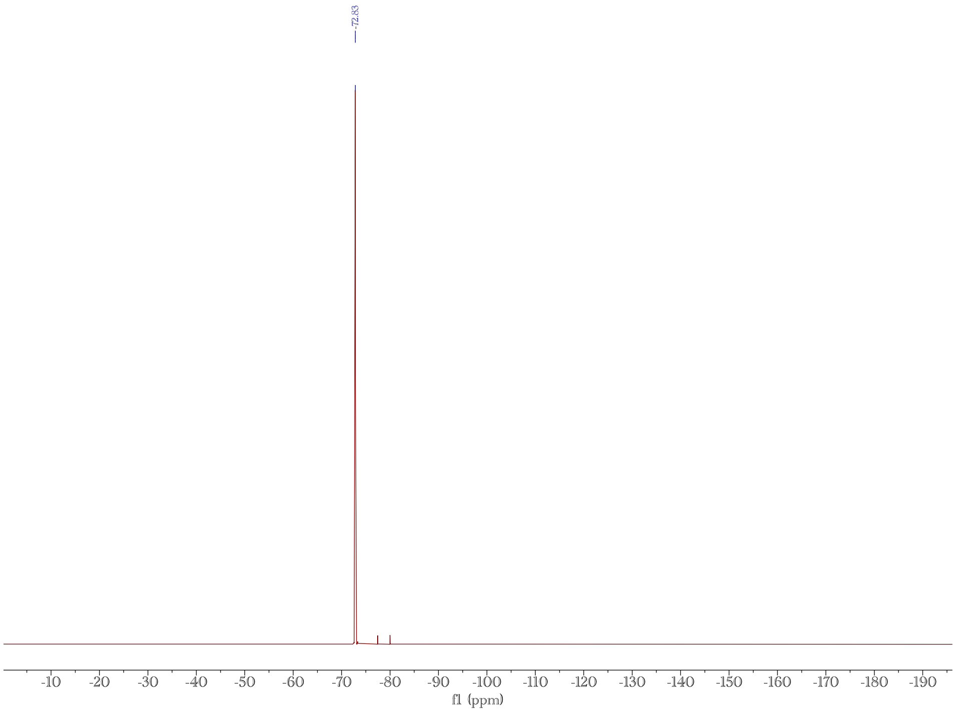

Figure S35. ^{19}F NMR spectrum of Compound 6.

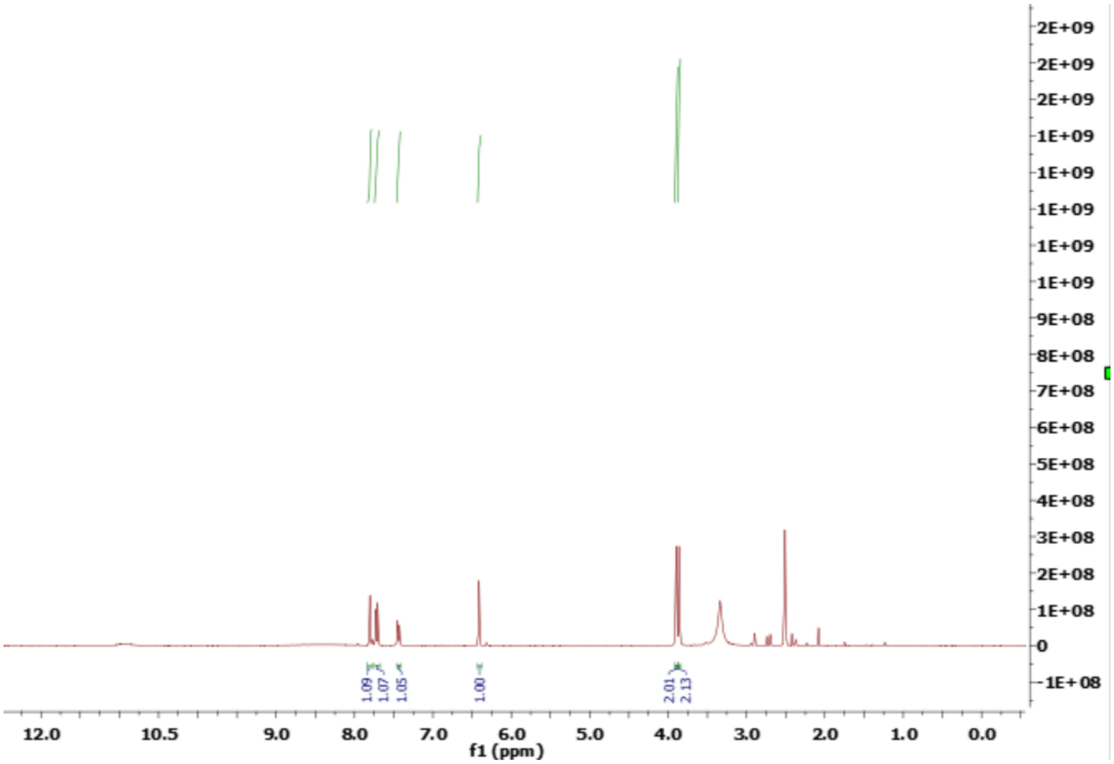

Figure S36. ^{1}H NMR spectrum of Glycine-7-amino-4-coumarinyl-acetic acid (Gly-ACA)

Table S1. The molecular weight of Ub and synthesized Ub Activity-based probes

Protein	Observed mass (Da)	Theoretical mass (Da)
Ub_{1-75}-Cys-6×His	9433.4	9433.7
Flag-Ub_{1-75}-Cys-6×His	10702.1	10702.0
Flag-Ub_{1-74}-Cys-6×His	10645.1	10645.4
UbPa	8543.6	8543.8
FlagUbPa	9812.2	9812.1
FlagUbMTC	9847.2	9847.1
FlagUbETC	9862.2	9862.1
FlagUbTFEGHC	9915.2	9915.5
UbACA	8765.7	8765.5

Table S2A. The molecular weight of DUBs tested and formed DUB-Probe complex

Deubiquitinases	MW of Deubiquitinases	MW of DUB-Probe Complex
USP2 CD	~41 kDa	~51 kDa
USP5	~96 kDa	~106 kDa
USP7 CD	~41 kDa	~51 kDa
USP8	~127kDa	~137 kDa
USP10	~90 kDa	~109 kDa
USP14	~68 kDa	~78 kDa
GST-USP15	~140 kDa	~150 kDa
USP25	~125 kDa	~135 kDa
CYLD	~135 kDa	~145 kDa
UCHL1	~25 kDa	~35 kDa
UCHL3	~25 kDa	~35 kDa
UCHL5	~37 kDa	~47 kDa
GST Ataxin-3	~70 kDa	~80 kDa
GST-JOSD2	~50 kDa	~60 kDa
OTUB1	~35 kDa	~45 kDa
OTUB2	~27 kDa	~37 kDa
OTULIN	~45 kDa	~55 kDa
Cezanne-OTUD7B	~45 kDa	~55 kDa
OTUD2	~38 kDa	~48 kDa
OTUD6B	~37 kDa	~47 kDa
GST-OTUD7C	~69 kDa	~79 kDa

Table S2B. The molecular weight of E1, E2, E3 enzymes tested and formed enzyme-probe complex

Enzymes	MW of Enzymes	MW of Enzyme-Probe Complex
UBE1	~118 kDa	~128 kDa
UbE2E2	~22 kDa	~32 kDa
Parkin	~52 kDa	~62 kDa
UbE3A	~100kDa	~110 kDa
ITCH	~110 kDa	~120 kDa

Table S3. Enzymes in the ubiquitin-proteasome pathway labelled by three ubiquitin azapeptide ester probes in HEK293T cell lysate pretreated with UbPa

Accession	Gene Symbol	Description
P09936	UCHL1	Ubiquitin carboxyl-terminal hydrolase isozyme L1
P15374	UCHL3	Ubiquitin carboxyl-terminal hydrolase isozyme L3
Q9Y5K5	UCHL5	Ubiquitin carboxyl-terminal hydrolase isozyme L5
P61077	UbE2D3	Ubiquitin-conjugating enzyme E2 D3
Q96FW1	OTUB1	Ubiquitin thioesterase OTUB1
Q8N6M0	OTUD6B	Deubiquitinase OTUD6B

Table S4. Other proteins identified in proteomics in HEK293T cell lysates pretreated with UbPa

Accession	Gene Symbol	Description
P62195	PSMC5	26S proteasome regulatory subunit
Q16186	ADRM1	Proteasomal ubiquitin receptor
P55036	PSMD4	26S proteasome non-ATPase regulatory subunit
P09012	SNRPA	U1 small nuclear ribonucleoprotein A
P28070	PSMB4	Proteasome subunit beta type-4
P20618	PSMB1	Proteasome subunit beta type-1
P25786	PSMA1	Proteasome subunit alpha type-1
P25787	PSMA2	Proteasome subunit alpha type-2
O14818	PSMA7	Proteasome subunit alpha type-7
Q96CT7	CCDC124	Coiled-coil domain containing protein
P08579	SNRPB2	U2 small nuclear ribonucleoprotein B
P62979	RPS27A	Ubiquitin 40S ribosomal protein S27A
P62987	UBA52	Ubiquitin 40S ribosomal protein L40
A2NJV5	IGHV2-29	IgG kappa variable 2-29
A0A0B4J1V2	IGHV2-26	IgG heavy variable 2-26
